# Maximizing the cost-effectiveness of cervical screening in the context of routine HPV vaccination by optimizing screening strategies with respect to vaccine uptake: a modeling analysis

**DOI:** 10.1186/s12916-023-02748-3

**Published:** 2023-02-10

**Authors:** Horace C. W. Choi, Kathy Leung, Karen K. L. Chan, Yuan Bai, Mark Jit, Joseph T. Wu

**Affiliations:** 1 School of Public Health, Li Ka Shing Faculty of Medicine, The University of Hong Kong, Patrick Manson Building (North Wing), 7 Sassoon Road, Hong Kong Special Administrative Region, China; 2 Laboratory of Data Discovery for Health Limited, Hong Kong Science Park, Hong Kong Special Administrative Region, China; 3 Department of Obstetrics and Gynaecology, Li Ka Shing Faculty of Medicine, The University of Hong Kong, Hong Kong Special Administrative Region, China; 4 Department of Infectious Disease Epidemiology, London School of Hygiene and Tropical Medicine, London, UK; 5 Modelling and Economics Unit, Public Health England, London, UK

**Keywords:** Cervical cancer screening, Cost-effectiveness analysis, Population-based screening program, HPV vaccination

## Abstract

**Background:**

Regarding primary and secondary cervical cancer prevention, the World Health Organization proposed the cervical cancer elimination strategy that requires countries to achieve 90% uptake of human papillomavirus (HPV) vaccines and 70% screening uptake. The optimal cervical screening strategy is likely different for unvaccinated and vaccinated cohorts upon national HPV immunization. However, health authorities typically only provide a one-size-fits-all recommendation for the general population. We aimed to evaluate the cost-effectiveness for determining the optimal screening strategies for vaccinated and unvaccinated cohorts.

**Methods:**

We considered the women population in Hong Kong which has a unique HPV infection and cervical cancer epidemiology compared to other regions in China and Asia. We used mathematical models which comprise a deterministic age-structured compartmental dynamic component and a stochastic individual-based cohort component to evaluate the cost-effectiveness of screening strategies for cervical screening. Following the recommendations in local guidelines in Hong Kong, we considered strategies that involved cytology, HPV testing, or co-testing as primary cervical screening. We also explored the impacts of adopting alternative de-intensified strategies for vaccinated cohorts. The 3-year cytology screening was used as the base comparator while no screening was also considered for vaccinated cohorts. Women’s lifetime life years, quality-adjusted life years, and costs of screening and treatment were estimated from the societal perspective based on the year 2022 and were discounted by 3% annually. Incremental cost-effectiveness ratios (ICERs) were compared to a willingness to pay (WTP) threshold of one gross domestic product per capita (US $47,792). Probabilistic and one-way sensitivity analyses were conducted.

**Results:**

Among unvaccinated cohorts, the strategy that adds reflex HPV to triage mild cytology abnormality generated more life years saved than cytology-only screening and could be a cost-effective alternative. Among vaccinated cohorts, when vaccine uptake was 85% (based on the uptake in 2022), all guideline-based strategies (including the cytology-only screening) had ICERs above the WTP threshold when compared with no screening if the vaccine-induced protection duration was 20 years or longer. Under the same conditions, HPV testing with genotyping triage had ICERs (compared with no screening) below the WTP threshold if the routine screening interval was lengthened to 10 and 15 years or screening was initiated at ages 30 and 35 years.

**Conclusions:**

HPV testing is a cost-effective alternative to cytology for vaccinated cohorts, and the associated optimal screening frequency depends on vaccine uptake. Health authorities should optimize screening recommendations by accounting for population vaccine uptake.

**Supplementary Information:**

The online version contains supplementary material available at 10.1186/s12916-023-02748-3.

## Background

Cervical cancer is one of the most common cancers among women, with more than 600,000 new cases and 340,000 cancer deaths in 2020 [1]. Regarding primary and secondary cervical cancer prevention, the World Health Organization (WHO) proposed that 90% of girls get fully vaccinated with human papillomavirus (HPV) vaccines by the age of 15 years and 70% of women get screened with a high-performance test (preferably an HPV DNA-based test) by age 35 and again by 45 years [2]. HPV vaccines are more than 90% efficacious against targeted oncogenic high-risk HPV (hrHPV) types; in particular, the second-generation nonavalent HPV (9vHPV) vaccine covers seven hrHPVs, which collectively cause more than 90% of cases of cervical cancer [3]. Over 100 countries have already included routine female HPV vaccination in their national immunization programs; however, vaccine uptake in most countries is well below 90% [4].

Upon vaccination, HPV prevalence in vaccinated cohorts drops through either direct vaccine-induced protection or indirect herd protection, with more substantial reductions in regions with higher vaccine uptake. With a lowered positive predictive value of screening, the benefit of frequent screening falls, and there is a greater risk of unnecessary referrals for colposcopy and unnecessary treatment of women with positive results [5, 6]. In populations where centralized healthcare systems are not mature and national immunization or screening programs are not yet implemented, the database for vaccination and screening records may not be linked well. On that occasion, it may not be easy to identify individuals’ vaccination status by the time when women attend cervical screening. Furthermore, from policymaking and administrative viewpoints, it may potentially be more feasible to adopt different cervical screening strategies for cohorts depending on whether they are of the ages eligible for vaccination due to the different risks of infection, rather than personal risks based on their vaccination status.

In countries such as Australia, Norway, and the UK that have commenced routine HPV vaccination for over a decade [4], the vaccinated cohorts have already reached or will soon reach the age of cervical screening, and updating screening recommendations is an urgent task. Most healthcare authorities provide screening guidelines for the entire population regardless of vaccination history. Recent literature has included discussions of the implementation of different cervical screening strategies per women’s vaccination status. Several modeling studies suggested that vaccinated women could be screened less frequently (when compared to unvaccinated women) for cervical screening remaining cost-effective on top of mass HPV vaccination [5, 7, 8]. In particular, the optimal number of lifetime screens may depend on the type of HPV vaccines received as well as population-specific disease epidemiology [9–11]. Most of these studies considered populations that have implemented mass HPV vaccination programs in the early phase when HPV vaccines were available on the market.

China has been suffering substantially from cervical cancer with more than 100,000 new cases annually (attributing to approximately 20% of the global incidence). Cervical screening uptake was low (< 30%) in China, and HPV vaccination has not yet been completely included in its national immunization program [12, 13]. To optimize healthcare resource utilization, our study aimed to evaluate the cost-effectiveness of cervical screening strategies for cohorts of eligible and non-eligible ages for vaccination upon the implementation of routine HPV immunization in the country. We considered the case of the Hong Kong population which has recently started a population-based HPV vaccination program. Hong Kong has a unique epidemiology of HPV prevalence and cervical cancer when compared to other regions in China. HPV prevalence in Hong Kong showed a major peak in younger ages (20–29 years) and a minor peak at older ages (approximately 50 or above) [14, 15]. Such a bimodal pattern was different from the observed HPV prevalence in China which showed a single peak at 35–54 years [16]. The cervical cancer incidence in Hong Kong was quite steady (at approximately 20 per 100,000 women) throughout the ages of 40 to 85 or above, with a small peak at ages 80–84 [17]. In contrast, cervical cancer incidence in China peaked at age 45–54 and then dropped as age increased [16]. Furthermore, the recently updated cervical screening guidelines in Hong Kong indicated several approaches to triage higher-risk women for further investigation (such as involving combinations of with/without HPV genotyping and cytology for HPV-positive screenees) [18, 19]. This may be worthwhile to compare the cost-effectiveness across different management, in addition to varying screening ages and frequencies. Despite the difference in epidemiology, we aimed to use our work to serve as a reference for an assessment framework for switching cervical screening strategies in the context of routine HPV vaccination for the Chinese population.

## Methods

### Model overview

We adopted our previously calibrated model of HPV vaccination and cervical cancer screening to estimate the costs and health outcomes of cervical screening strategies for Hong Kong [20]. Briefly, the model comprises (i) a deterministic age-structured compartmental dynamic model for simulating the heterosexual transmission of hrHPVs and (ii) a stochastic individual-based cohort model for simulating the development of cervical cancer over the lifetime of each female [20]. Both dynamic and stochastic components were based on the same natural history model of cervical cancer development that includes health states such as HPV infection, precancerous cervical intraepithelial neoplasia (CIN), and preclinical asymptomatic and clinical symptomatic cancers. Regarding HPV infection, we grouped hrHPVs into four classes: (i) HPV-16; (ii) HPV-18; (iii) HPV-OV (for “other vaccine types”), which comprises the other five hrHPVs targeted by the 9vHPV vaccine, namely, HPV-31, 33, 45, 52, and 58; and (iv) HPV-NV, which comprises all the non-vaccine hrHPVs (i.e., HPV-26, 35, 39, 51, 53, 56, 59, 66, 67, 68, 69, 73, and 82) [14]. The dynamic model was used to infer the model parameters using empirical data from the prevaccination era and then to estimate herd effects after routine female adolescent HPV vaccination had begun. The age-specific force of infection from the dynamic model was used in the stochastic individual-based model to simulate cervical cancer incidence for each birth cohort. The cohort model simulated cervical screening practices and treatments according to the guidelines issued by the Hong Kong College of Obstetricians and Gynaecologists (HKCOG; 2016) [19]. A monthly stepsize was used, and individuals in the population up were simulated up to age 85 years.

Based on the natural history model, the inferring parameters include transition rates between health states and the assortativeness variables in the formation of sexual partnerships between females and males. We assumed that respective parameters that were related to HPV infection were the same in both genders. We estimated the jointly correlated parameters by calibrating the model to empirical data. We first simulated the natural history model based on a given parameter set. We then compared the similarity between the modeled and observed fitting outcomes which included local age-specific HPV prevalence and cervical cancer incidence [14, 15, 17]. The process was iterated to identify parameter sets that showed good calibration (i.e., high similarity) to the fitting targets. We used the Markov chain Monte Carlo approach to update the parameters when calibrating the model. This technique of parameter inference that synthesizes the disease’s natural history model and multiple empirical targets has also been adopted in building microsimulation models for cervical cancer screening and vaccination and screening of colorectal and breast cancers in overseas studies [21–23]. Additional file 1: Supplementary Information lists more details on model description (pages 2-6; Additional file 1: Fig. S1 and Table S1) [14, 21, 24–34], parameterization, and calibration (pages 7–11; Additional file 1: Figs. S2-S3, Tables S2-S3) [14, 15, 17, 20, 22, 23, 33, 35–42].

### HPV vaccination

Following the most recent statistics, we set the 2-dose vaccine uptake at 85% as the base case scenario and also explored scenarios with lower vaccine uptakes of 75%, 50%, and 25% [43]. The latest literature suggests that first-generation HPV (bivalent [2vHPV] and quadrivalent [4vHPV]) vaccines remain protective beyond 10 years, with no indication of secondary vaccine failure in the cohorts who received 4vHPV vaccines more than 15 years ago in 2006 [44, 45]. As such, we considered three scenarios, namely lifelong, 30-year, and 20-year, for the protection durations induced by the 9vHPV vaccines. We assumed lifelong vaccine-induced protection as the base case scenario. The vaccine efficacy of the 9vHPV vaccine against HPV-16, HPV-18, and HPV-OV was based on 9vHPV vaccine trial data and local HPV epidemiology [3, 14, 46]. Additional file 1 lists more related details (page 12) [3, 14, 43, 46–50].

### Cervical screening

**Fig. 1:**
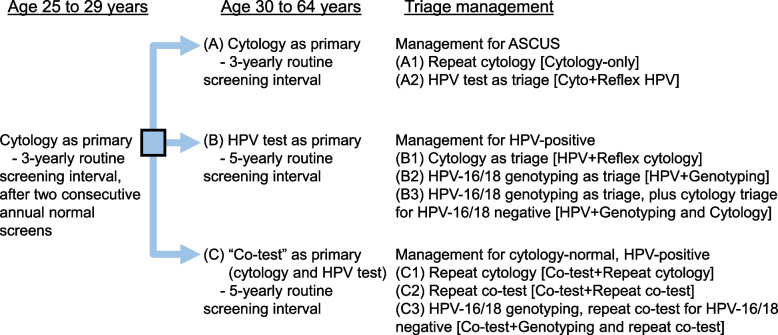
Cervical screening algorithms that are currently recommended in Hong Kong. ASCUS, atypical squamous cells of undetermined significance

We referred to the screening strategies that are recommended by the latest local screening guidelines by the Cancer Expert Working Group and the HKCOG guidelines (Fig. 1) [19, 51]. We considered strategies that use (A) cytology, (B) high-risk HPV DNA testing (HPV testing), or (C) “co-testing” (i.e., combining cytology and HPV testing) as the primary test modality. Following the HKCOG guidelines, we assumed that the routine screening interval was 3 years for (A) primary cytology and 5 years for (B) primary HPV testing and (C) co-testing as the primary screening method. Screening would start at age 25 with primary cytology for all screening strategies examined. Under strategies B1–B3 and C1–C3, HPV testing and co-testing at age 30 were the primary testing strategies, respectively. Additional file 1: Supplementary Information provides more relevant details (pages 12–16; Additional file 1: Fig. S4) [18, 19, 51–56].

The analysis was performed separately for (a) cohorts who were too old to be eligible for the routine HPV vaccination program (i.e., no longer studying in primary schools, usually aged 13 years or above) when it commenced in 2019 (the unvaccinated cohorts) and (b) the first ten cohorts who had the opportunity to receive HPV vaccination via the routine program (the vaccinated cohorts). That is, the unvaccinated cohorts included women aged 16 to 64 years, and the vaccinated cohorts included those aged 6 to 15 years in 2022. According to the findings in local surveys, we assumed that 70% of eligible women would undergo cervical screening at each visit [18]. We also assumed that all women who have initiated cervical screening would follow recommended screening visits and clinical appointments for evaluating the maximum impacts of screening [8].

The current HKCOG guidelines were developed before the routine HPV vaccination program commenced in 2019 [19]. Studies outside Hong Kong suggested that in the era of HPV vaccination, women may perceive a lower risk of HPV infection [5, 8]. The current frequency of routine screening in Hong Kong may no longer be cost-effective among vaccinated cohorts. Thus, we further explored the cost-effectiveness of de-intensified strategies that (a) involve longer routine screening intervals (e.g., every 10 or 15 years), (b) initiate screening at older ages (e.g., at 30 or 35 years), or (c) stop screening after a predetermined number of normal screens during a lifetime for vaccinated cohorts.

We adopted the findings reported in the meta-analyses for the sensitivity and specificity of cytology and HPV testing (Additional file 1: Table S4) [19, 57–61].

### Cost-effectiveness analysis (CEA)

We estimated the incremental cost-effectiveness of each screening option using a societal perspective. We consulted local oncologists and gynecologists for the standard management procedures of cervical precancerous lesions and cervical cancers and then referred to corresponding treatment charges for private services provided in public hospitals by the Hospital Authority [62]. The Hospital Authority manages all public hospitals which accounted for over 90% of inpatient care in Hong Kong, and we assumed that the costing parameters could be generalizable in Hong Kong [63]. The private charges excluded subsidies from the government, and we assumed that the public hospitals make these charges for covering their costs instead of making a profit. We used life year (LY) and quality-adjusted life year (QALY) as the metrics for quantifying health outcomes and considered LY as the primary metric because it was more commonly used in international CEAs on cervical screening [5, 6, 8, 64]. When calculating QALYs, we adopted health utility weights for screening and cancer outcomes based on international studies due to the lack of local data [6]. Both cost and health benefits were calculated and discounted at 3% annually, starting from 2022. The costs were denominated in US dollars (US $1 = HK $7.8). Table 1 lists the cost and health utility parameters we used in the analysis, with more details in Additional file 1: Supplementary Information (page 18) [6, 19, 20, 25, 31, 62, 63, 65–75].

**Table 1:** Cost and health utility parameters that are used in the analysis

**Cost items**	**Distribution (US $)**	**References**
Cytology test	*T*(37.2, 111.5, 74.4)	[62]
HPV test	*T*(65.4, 130.8, 87.2)	[62]
Colposcopy + biopsy	*T*(552.6, 1157.7, 855.1)	[62]
Treatment for CIN2 or CIN3: loop electro-surgical excision procedure (LEEP)	*N*(2164.1, 541.0)	[62]
Treatment for stage I cervical cancer: Wertheim’s hysterectomy	*N*(15,510.3, 3877.6)	[62]
Treatment for stage II-III cervical cancer: radiotherapy + chemotherapy + brachytherapy	*N*(48,212.2, 12,053.1)	[62]
Treatment for stage IV cervical cancer: radiotherapy + chemotherapy	*N*(24,902.3, 6225.6)	[62]
Palliative care hospitalization (per day)	*T*(567.9, 852.6, 710.3)	[62]
Staff cost for screening	*N*(25.6, 6.4)	[62]
Staff cost for treatment of precancerous lesions	*N*(109.0, 27.3)	[62]
Time cost (half day)	*N*(31.3, 7.8)	[65]
Transportation	*N*(6.4, 1.6)	[66, 67]
**Health outcomes**	**Distribution**	**References**
Utility loss per episode of screen results		
Negative cytology/negative HPV test	*T*(0.00002, 0.00023, 0.0001)	[6]
ASCUS	*T*(0.00023, 0.002, 0.0011)	[6]
Positive HPV test	*T*(0.00023, 0.0089, 0.004)	[6]
Normal colposcopy	*T*(0.0015, 0.04, 0.0147)	[6]
LSIL/CIN1	*T*(0.005, 0.11, 0.0618)	[6]
CIN23	*T*(0.003, 0.13, 0.0783)	[6]
Quality of life weight during and post-treatment of cervical cancer, during treatment (6 months or until death)		
Stage I	*T*(0.49, 0.81, 0.705*)*	[6, 68]
Stage II	*T*(0.42, 0.67, 0.615)	[6, 68]
Stage III	*T*(0.42, 0.70, 0.56)	[68]
Stage IV	*T*(0.36, 0.60, 0.48)	[68]
Post-treatment (4.5 years or until death)		
Stage I	*T*(0.73, 0.99, 0.97)	[6, 68]
Stage II	*T*(0.68, 0.98, 0.935)	[6, 68]
Stage III	*T*(0.68, 0.98, 0.935)	[6, 68]
Stage IV	*T*(0.47, 0.969, 0.795)	[6, 68]

*N*(*a*, *b*) denotes a normal distribution with mean *a* and standard deviation *b*. A coefficient of variation of 0.25 was considered when the standard deviation was unavailable for the normal distribution. *T*(*a*, *b*, *c*) denotes triangular distribution that ranges from *a* to *b* with mode *c*. Hospitalization costs were included for cancer treatment whenever necessary

*Abbreviations*: *ASCUS* atypical squamous cells of undetermined significance, *CIN* cervical intraepithelial neoplasia, *LSIL* low-grade squamous intraepithelial lesion

We conducted probabilistic sensitivity analysis (PSA) to account for parameter uncertainty. A total of 10,000 combinations of parameters were sampled with Latin hypercube sampling (pages 18–19, Additional file 1: Supplementary Information). We calculated the incremental cost-effectiveness ratio (ICER), which is defined as the incremental cost divided by the incremental health outcome, when comparing the two strategies. The incremental cost and health outcome were estimated as the difference in the mean cost and health outcome based on the PSA for the corresponding strategies, respectively. There is no official willingness to pay (WTP) threshold for CEAs in Hong Kong, and the WHO no longer recommends directly correlating the threshold with the gross domestic product per capita (GDPpc). Instead, we set the threshold at one GDPpc based on previous studies conducted there [20, 72]. The average GDPpc in Hong Kong during 2017–2021 was US $47,792 [76]. We considered the 3-year cytology screening (strategy A1), which was recommended before the recent update in 2021, as the base comparator when comparing the impacts of alternative strategies. When sorting strategies on the frontier for incremental CEAs, the strategy with the lowest cost-effectiveness ratio with respect to the scenario of no screening would be ranked the first non-dominated strategy in the league table and then followed by strategies with the lowest ICERs compared with the previous non-dominated strategies [66]. Additional file 1: Supplementary Information provides more information on the analysis (page 19) [20, 66, 76–81].

We performed a one-way sensitivity analysis (OWSA) on selected strategy comparisons (Additional file 1: Supplementary Information; page 19) [82, 83]. We tested annual discount rates at 0% (i.e., undiscounted) and 6% as well as screening participation rates at 50% and 100% in the sensitivity analysis. Furthermore, we included a scenario analysis which assumed that males have a faster HPV clearance and shorter natural immunity, based on the observations in some clinical studies (page 19, Additional file 1: Supplementary Information) [21, 24, 32, 84–87].

We followed the Consolidated Health Economic Evaluation Reporting Standards 2022 (CHEERS 2022) statement and HPV-FRAME checklist for reporting health economic and HPV-related cancer control evaluations, respectively (Additional file 2; CHEERS 2022 and HPV-FRAME checklists) [88, 89].

## Results

### Cohorts without a routine vaccination program (unvaccinated cohorts)

**Fig. 2:**
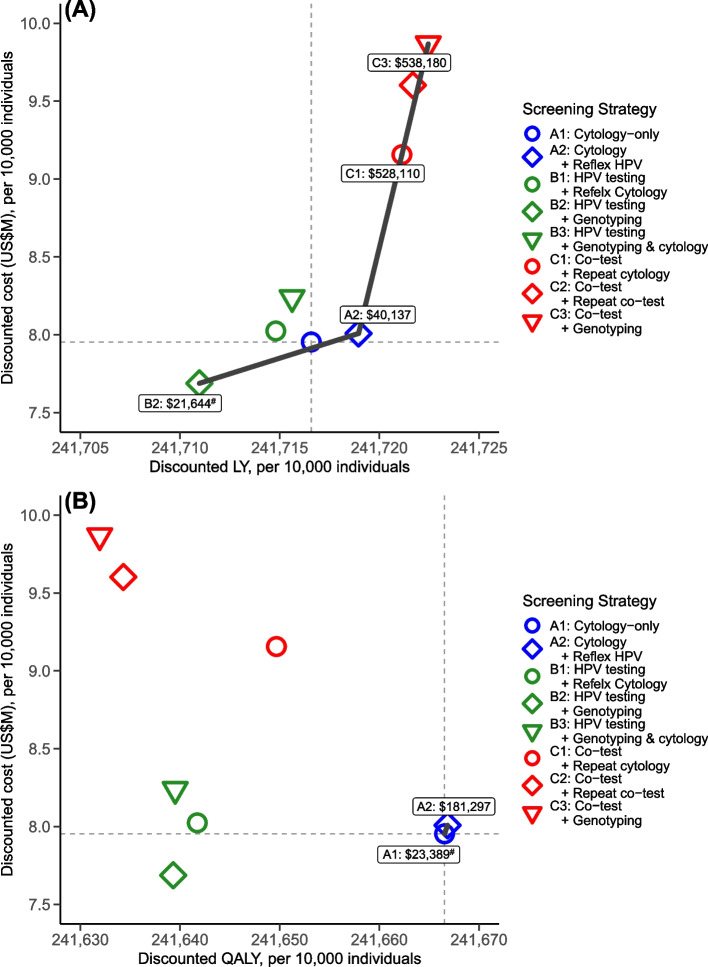
Cost-effectiveness of guideline-based screening strategies for cohorts without a routine vaccination program (unvaccinated cohorts). Using **A** life years (LYs) and **B** quality-adjusted life years (QALYs) as metrics for quantifying health outcomes. *Note*: The thick black lines are the cost-effectiveness frontiers, with the numbers in round-cornered rectangles representing the incremental cost-effectiveness ratios compared to “no screening” (for the first non-dominated strategy, denoted with ^#^) or the previous non-dominated strategy

In the base case scenario where vaccine uptake was 85% and vaccine protection was lifelong, strategies B1–3 that adopt HPV testing as primary screening with a 5-year regular screening interval generated fewer LYs/QALYs than the 3-year cytology screening (strategy A1); among which, strategy B2 (HPV + genotyping) was less costly than A1 (Additional file 1: Table S5). If LYs were used as the metric for health outcomes, strategy B2 incurred a lower ICER than A1, when both were compared with no screening (US $21,644 for B2 vs US $22,239 for A1 per LY gained), suggesting that B2 was the first non-dominated strategy among the guideline-based strategies evaluated. Strategy A2 (cytology + reflex HPV testing) incurred a lower ICER than A1 when both were compared with B2 (i.e., A2 dominated A1; Additional file 1: Table S5). Strategy A2 was the next most cost-effective strategy with an ICER of US $40,137 per LY gained (Fig. 2). The remaining strategies were either dominated or associated with ICERs above 3 times the WTP threshold. If QALYs were used as the metric for health outcomes instead, strategy A1 was the most cost-effective strategy, with an ICER of US $23,389 per QALY gained compared with no screening (Fig. 2). Switching to the next most cost-effective strategy, namely, strategy A2, would incur an ICER of US $181,297 per QALY gained. The remaining strategies were dominated.

The comparative cost-effectiveness of the optimal strategies for unvaccinated cohorts was not sensitive to vaccine uptake and duration of vaccine protection among the unvaccinated cohorts (Additional file 1: Table S6). The conclusions of the comparative cost-effectiveness when varying the annual discount rate to 0% and 6% were similar to the scenario of a 3% annual discount rate (Additional file 1: Table S7), except that when QALY was the metric for quantifying health outcomes, the estimated ICER for A1 (cytology-only) vs no screening became not cost-effective and exceeded the WTP threshold by 11%. The OWSA suggested that the estimated ICERs were more sensitive to test performance, such as the specificity of the cytology and/or HPV test and the sensitivity of detecting CIN (Additional file 1: Fig. S5). The estimated ICERs for switching to A2 (cytology + reflex HPV) from A1 (cytology-only) and adopting A1 compared with no screening remained below the WTP threshold when LY and QALY were the metrics for health outcomes, respectively. The detailed findings of sensitivity analyses are presented in Additional file 1: Supplementary Information (pages 21–26; Additional file 1: Tables S5-S8, Fig. S5).

### Cohorts with the opportunity to receive routine vaccination (vaccinated cohorts)

**Fig. 3:**
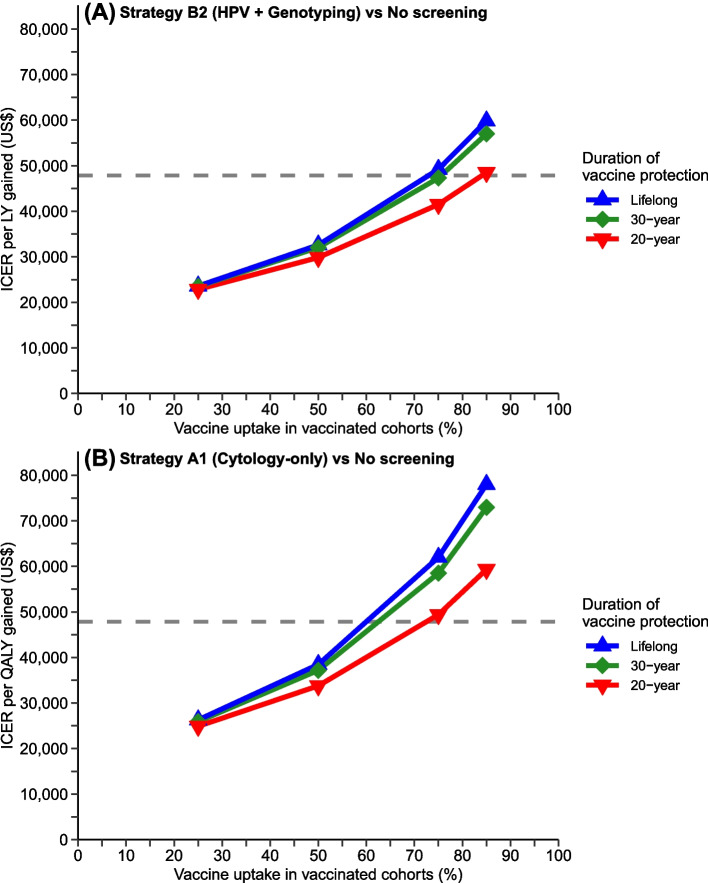
Incremental cost-effectiveness ratios (ICERs) of the most cost-effective guideline-based screening strategy across scenarios of vaccine uptake and duration of vaccine protection for cohorts in the routine vaccination program (vaccinated cohorts). **A** Using life years (LYs) as metrics for health outcomes. Comparing B2 (HPV + genotyping) vs no screening. **B** Using quality-adjusted life years (QALYs) as metrics for health outcomes. Comparing A1 (cytology-only) vs no screening. *Note*: The gray dashed lines represent the willingness to pay threshold at 1 GDPpc (US $47,792)

The comparative cost-effectiveness of the evaluated strategies for the vaccinated cohorts was sensitive to vaccine uptake and duration of vaccine protection among the vaccinated cohorts. In the base case scenario where vaccine uptake was 85% and vaccine protection was lifelong, the ICERs of all guideline-based strategies with recommended routine screening intervals, including the 3-year cytology screening (strategy A1), exceeded the WTP threshold when compared with no screening (Additional file 1: Table S9). Strategy B2 (HPV testing + genotyping) and strategy A1 (cytology-only) had the lowest ICERs at US $59,863 per LY gained and US $78,003 per QALY gained when compared with no screening, respectively. The ICERs of strategies B2 and A1 remained above the WTP threshold for using LYs and QALYs as the metrics for health outcomes, respectively, when the vaccine uptake of immunization programs was 75% or above among the evaluated scenarios, unless the vaccine uptake was 50% or below (Fig. 3 and Additional file 1: Table S10).

If the annual discount rate was 0% (i.e., undiscounted), strategy B2 was the most cost-effective in the settings where LY and QALY were the metrics for health outcomes, with estimated ICERs below US $27,000 when compared with no screening (Additional file 1: Table S11). Continuing to adopt strategy A1 (cytology-only) was less effective (but more costly) or incurred an ICER exceeding the WTP threshold when compared with B2. If the discount rate was 6%, the estimated ICERs were greater than at least 2.8 times the WTP threshold even for the most cost-effective strategies compared with no screening (Additional file 1: Table S11). The OWSA suggested that the estimated ICERs were more sensitive to test performance, such as the specificity of cytology and/or HPV test and the sensitivity of detecting CIN, as well as the cost of cancer treatment (Additional file 1: Fig. S6). Under OWSA, the estimated ICERs of strategies B2 and A1 (which are the most optimal strategies compared with no screening when LY and QALY were the metrics for health outcomes, respectively) remained above the WTP threshold. In the scenario analysis that assumed the HPV clearance rate and waning rate of natural immunity were faster in males, the incremental CEA was similar to the original findings, under the setting of substantial vaccination impacts with 85% vaccine uptake and lifelong vaccine protection (Additional file 1: Table S12). The detailed findings of the sensitivity analysis are presented in Additional file 1: Supplementary Information (pages 27–33; Additional file 1: Tables S9-S13, Fig. S6).

**Fig. 4:**
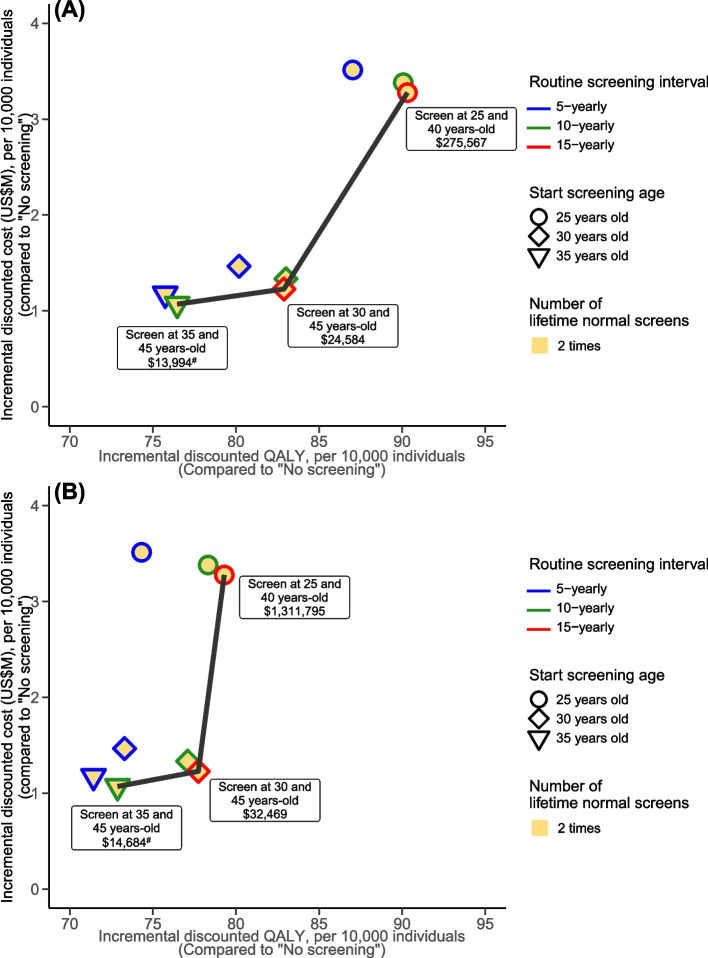
Cost-effectiveness of variants of strategy B2 (HPV + genotyping) that meet WHO’s proposal of screening women between the ages of 35 and 45 years for cohorts implemented with the routine vaccination program (vaccinated cohorts). Using **A** life years (LYs) and **B** quality-adjusted life years (QALYs) as metrics for quantifying health outcomes. ^a^Assume that 9vHPV vaccines provided lifelong protection and the vaccine uptake was 85%. ^b^Following the HKCOG guidelines, for strategy B2 (HPV + genotyping), women would start screening with cytology at the age of 25 years and then switch to the primary HPV test after 30 years. For variants of strategy B2 that start screening age at 30 and 35 years, women would directly undergo primary HPV testing at 30 and 35 years, respectively, without the prior cytology screening at age 25–29 years. ^c^The *x*- and *y*-axes represent the incremental discounted cost and LY/QALY compared with “no screening.” ^d^The black thick lines are the cost-effectiveness frontiers, with the numbers in round-cornered rectangles representing the ICERs compared to “no screening” (for the first non-dominated strategy, denoted with ^#^) or the previous non-dominated strategy

To further assess the cost-effectiveness of cervical screening in vaccinated cohorts with high vaccine uptake, we examined de-intensified strategies that use HPV testing as the primary screening tool per the WHO’s recommendation. We considered variants of strategy B2 (HPV testing + genotyping) which incurred the lowest ICERs among the primary HPV testing strategies (i.e., strategies B1–B3) indicated in the local guidelines. Figure 4 presents the cost-effectiveness of variants of strategy B2 (HPV testing + genotyping) that meet the WHO’s proposal of screening women between ages 35 and 45 years for vaccinated cohorts, under the base case scenario of 85% vaccine uptake and lifelong vaccine protection. Assuming that women stopped screening after obtaining two normal HPV test results (as the minimum requirement per the WHO’s proposal), the ICER of a variant of strategy B2 that initiated cervical screening at age 35 with another screen 10 years later was US $13,994 per LY gained and US $14,682 per QALY gained when compared with no screening. Screening women at the ages of 30 and 45 was the next non-dominated strategy; the ICERs were US $24,583 per LY gained and US $32,469 per QALY gained when compared with screening women at the ages of 35 and 45. Other evaluated potential alternatives, including strategies that relaxed the restriction that women stop screening after obtaining two normal HPV test results (i.e., women may continue to screen until age 65), were either dominated or associated with an ICER above 3 times the WTP threshold (Additional file 1: Table S14 and S15).

## Discussion

We evaluated the cost-effectiveness of guideline-based cervical screening strategies and potential alternatives for the situation in Hong Kong, where the vaccine uptake (among cohorts via the immunization program) and screening uptake are close to the WHO’s proposed targets while the public health impacts of incorporating hrHPV testing as a primary tool in cervical screening in the era of HPV vaccination are currently lacking [2, 19, 51]. Our findings demonstrate that the optimal screening strategy is different for cohorts that are of eligible and non-eligible ages for the HPV vaccination program (i.e., vaccinated and unvaccinated cohorts, respectively) when vaccine uptake is high. In line with the WHO’s recommendation of using HPV testing as the primary screening tool [2], the HKCOG guidelines stated three approaches to triage HPV-positive cases with 5-year regular screening intervals (i.e., strategies B1–3). For unvaccinated cohorts, our findings suggested that HPV-primary screening strategies generated fewer LY gained than the 3-year cytology screening (strategy A1). Instead, strategy A2 which uses a reflex HPV test to triage mild cytology abnormality (atypical squamous cells of undetermined significance, ASCUS) may be considered a cost-effective alternative to A1 (cytology-only) with more LY gained.

For vaccinated cohorts, the comparative cost-effectiveness of screening strategies depends on vaccine uptake and duration of vaccine protection. When the impact of the immunization program is high (e.g., when vaccine uptake is high or vaccine protection is long), the marginal benefit of screening is lower, and the corresponding ICERs of screening strategies are higher. Across the scenarios considered here, vaccine impact is the highest when vaccine uptake is 85% (the status quo) and vaccine protection is lifelong. In this scenario, all evaluated screening strategies with guideline-based routine screening intervals, including the 3-year cytology screening strategy (A1), are not cost-effective when compared with no screening. This suggests the need to adopt less-intensive strategies for screening to remain a cost-effective add-on in the scenario of high vaccine uptake. However, when vaccine uptake is low to moderate (e.g., at 50% or below), strategies with guideline-based routine screening intervals may achieve ICERs below the WTP threshold, suggesting that a one-size-fits-all screening strategy may be used for both unvaccinated and vaccinated cohorts.

The WHO recommends the target of 70% female screening coverage (preferably with HPV testing) at least twice by the age of 35 and another test by the age of 45 [2]. The triage approaches used in national screening programs vary across different countries. For example, Public Health England recommends using cytology tests as triage, while genotyping of HPV-16/18 is adopted in Australia [90, 91]. We evaluated de-intensified variants of strategy B2 (HPV + genotyping) which is the most cost-effective among the three primary HPV testing strategies stated in the HKCOG guidelines. Our results suggest that for vaccinated cohorts, HPV testing with genotyping triage, by which women were screened twice either at ages 35 and 45 (10 years apart) or at ages 30 and 45 (15 years apart), achieved ICERs below US $16,000 per LY or QALY gained when compared with no screening. In particular, initiating screening at a younger age of 30 years with a longer interval of 15 years for the second routine screen may generate more LYs and QALYs gained while remaining cost-effective with an ICER under the WTP threshold when compared with screening women at ages 35 and 45. This demonstrates the feasibility of exploring alternatives that provide better value for money in resource allocation. However, the safety and compliance of the alternative strategies should also be monitored regularly.

To follow the status quo policy implementation in Hong Kong, we evaluated the cost-effectiveness of cervical screening in the context of female-only HPV vaccination and did not consider the case of a gender-neutral vaccination. In Hong Kong, the acceptance of HPV vaccination among adolescent male students was low. In a recent local survey, only 23% of the responded male students aged 18–26 years found HPV vaccination in males to be acceptable [92]. An overseas comparative modeling study predicted that if a 90% vaccine uptake among females was achieved, vaccine-targeted HPV types could almost be eliminated, and the corresponding incremental impact on reducing cervical cancer was limited for additionally vaccinating males [93]. Health economic studies also suggested that if HPV vaccination coverage among females was high (e.g., above 75%), including males in the vaccination was likely not cost-effective when compared with vaccinating females only [94]. Furthermore, given the supply shortage of HPV vaccines, the Strategic Advisory Group of Experts on Immunization of the WHO recommended temporarily pausing and lowering the priority of male vaccination [95, 96]. Therefore, we did not include the scenario of a gender-neutral HPV vaccination because this is less likely to be implemented in Hong Kong shortly. However, the impact of cervical screening in the context of vaccinating both genders would be worthwhile to be considered if the policy implementation changes in the future, particularly when the acceptance of male vaccination increases substantially.

Our study has some limitations. First, we assumed one screening strategy for both vaccinated and unvaccinated women in the vaccinated cohorts. Naber et al. reported that when herd protection is over 50% (i.e., infections are reduced by over 50% among unvaccinated women in vaccinated age cohorts), a shorter screening interval (e.g., a strategy that is optimized for prevaccination scenarios) for unvaccinated individuals in vaccinated cohorts might not be cost-effective [97]. That is, in cohorts with high vaccine uptake, such as in Hong Kong, the UK, and Australia where over 75% of schoolgirls are vaccinated via routine immunization programs, there is little advantage in conditioning screening strategies on women’s vaccination status. Therefore, we evaluated the impacts of cervical screening (by population-level vaccine uptake) in the cohorts regardless of individual vaccination status.

Furthermore, based on current data from Hong Kong, we assumed vaccine uptake to be 85% in the base case. We did not consider the case of 90% uptake, which the WHO recommends as a target, because only a few countries (e.g., Norway) have achieved such a high uptake [4]. Additionally, we considered the scenario of using 9vHPV vaccines in the immunization program, rather than 2vHPV and 4vHPV vaccines which have been used in some countries. Nevertheless, our conclusions about stratifying screening strategies per vaccination cohort likely remain valid as long as vaccine uptake exceeds 50% and when the 2vHPV and 4VHPV vaccines also provide high efficacy against HPV-16/18, which collectively contributes to 70% of cervical cancer cases.

Similar to other HPV modeling studies, we assumed that parameters that are related to HPV infection were the same in both genders [21, 24, 32]. Some studies suggested that clearance of HPV infection among males was comparable to that among females [84, 85], while some clinical findings observed that the time to clearance of HPV infection was shorter in males [87, 98]. On the other hand, some studies indicated that the natural immunity following recovery from HPV infection may be less persistent in males than that in females [86]. HPV prevalence is affected by multiple parameters relating to the natural history of HPV infection and sexual behavior. The HPV prevalence curve among males was more stable over older ages when compared to that among females which generally peaked at younger ages and declined afterwards [99, 100]. The scenario analysis (which assumed a faster HPV clearance rate and waning rate of natural immunity among males) suggested that under the setting of high vaccination impacts, the respective incremental CEA was comparable to the original findings which assumed that natural history parameters relating to HPV infection were the same in both genders (Additional file 1: Table S12). Moreover, in line with other studies on cervical screening, our study concludes that a longer screening interval could be adopted in vaccinated female cohorts when the vaccine uptake was high (e.g., over 75%) [5, 8, 11]. Nevertheless, the respective impacts of the gender-specific assumptions for the natural history parameters regarding HPV infection on the estimation of HPV burden and HPV vaccination may be worthwhile for further investigation.

Last, we estimated the model parameters by fitting simulated population-level HPV prevalence and cancer incidence to different empirical data sources [14, 15, 17]. This method has also been adopted for developing simulation models in colorectal and breast cancer screening [22, 23]. Using individual-level data from organized screening programs or longitudinal trials that trace the changes in health states across time horizons could be alternative approaches to estimating disease transition parameters [101]. In Hong Kong, cervical cytology has been used as the primary modality in cervical screening for decades. The use of HPV testing in cervical screening was introduced in the recent guidelines in 2016 [19]. To our knowledge, longitudinal data on HPV prevalence and incidence of precancerous CINs are not publicly available at the moment in Hong Kong. Therefore, we did not include individual-level data when inferring parameters in this study.

## Conclusions

When following the WHO’s targets to eliminate cervical cancer, high uptake of HPV vaccination is likely observed. For vaccinated cohorts, adopting de-intensified strategies with a reduced expected number of lifetime screens for HPV-based screening may be cost-effective if vaccine uptake of routine vaccination is 75% or above. To maximize the cost-effectiveness of cervical screening, health authorities should explicitly propose screening recommendations that are tailored separately with respect to birth cohorts involved in routine vaccination programs and base these recommendations on the actual vaccine uptake attained.

## Supplementary Information

**Additional file 1: Supplementary Information.** This file provides additional information on model description, parameterization and calibration, settings on cervical screening and HPV vaccination used in the study, extra data for performing the cost-effectiveness analysis, and the supplementary results on sensitivity analyses. **Table S1.** The distribution of individuals by the level of sexual activity and age group. **Table S2.** Age distribution and mortality rate in Hong Kong. **Table S3.** Posterior distributions of inferred parameters. **Table S4.** Test performance parameters of cytology and HPV test. **Table S5.** Cost-effectiveness of guidelines-based screening strategies for unvaccinated cohorts. **Table S6.** ICERs of the most cost-effective screening strategy by vaccine uptake and duration of vaccine protection for unvaccinated cohorts. **Table S7.** Cost-effectiveness of screening strategies by annual discount rates for unvaccinated cohorts. **Table S8.** Sensitivity analysis on the estimated costs and health outcomes for unvaccinated cohorts by screen uptake. **Table S9.** Cost-effectiveness of guidelines-based screening strategies for vaccinated cohorts. **Table S10.** ICERs of the most cost-effective screening strategy by vaccine uptake and duration of vaccine protection for vaccinated cohorts. **Table S11.** Cost-effectiveness of screening strategies by annual discount rates for vaccinated cohorts. **Table S12.** Cost-effectiveness of screening strategies for vaccinated cohorts, with the assumption of faster HPV clearance rate and waning rate of natural immunity in males. **Table S13.** Sensitivity analysis of the estimated cost and health outcomes of screening strategies for vaccinated cohorts by screening uptake. **Table S14.** One-way sensitivity analysis on the cost-effectiveness of variants of strategy HPV + Genotyping for vaccinated cohorts. **Table S15.** Cost-effectiveness of variants of strategy HPV + Genotyping for vaccinated cohorts. **Fig. S1.** Schematic of the natural history model for high-risk HPV infection and cervical cancer. **Fig. S2.** Comparison of empirical data and the fitted model. **Fig. S3.** Trace plots and the posterior distributions of fitted parameters. **Fig. S4.** Schematic of evaluated cervical screening algorithms. **Fig. S5.** One-way sensitivity analysis of ICER for unvaccinated cohorts. **Fig. S6.** One-way sensitivity analysis of ICER for vaccinated cohorts.

**Additional file 2: **CHEERS 2022 and HPV-FRAME checklists.

## Data Availability

The data generating the findings of this article are included within the article and its additional file.

## Abbreviations

2vHPV: Bivalent human papillomavirus vaccine
4vHPV: Quadrivalent human papillomavirus vaccine
9vHPV: Nonavalent human papillomavirus vaccine
ASCUS: Atypical squamous cells of undetermined significance
CEA: Cost-effectiveness analysis
CIN: Cervical intraepithelial neoplasia
GDPpc: Gross domestic product per capita
HKCOG: Hong Kong College of Obstetricians and Gynaecologists
HPV: Human papillomavirus
HPV-NV: Non-vaccine human papillomavirus types
HPV-OV: Other vaccine human papillomavirus types
hrHPV: High-risk human papillomavirus
ICER: Incremental cost-effectiveness ratio
LEEP: Loop electro-surgical excision procedure
LSIL: Low-grade squamous intraepithelial lesion
LY: Life year
OWSA: One-way sensitivity analysis
PSA: Probabilistic sensitivity analysis
QALY: Quality-adjusted life year
WHO: World Health Organization
WTP: Willingness to pay
