# Supplementary material for: Maximizing the cost-effectiveness of cervical screening in the context of routine HPV vaccination by optimizing screening strategies with respect to vaccine uptake: a modeling analysis
Source: BMC Med. 2023 Feb 10;21:48. doi: 10.1186/s12916-023-02748-3 (PMC9921628; doi:10.1186/s12916-023-02748-3)
Supplement: Supplementary file 1 — Additional file 1: Supplementary Information. This file provides additional information on model description, parameterization and calibration, settings on cervical screening and HPV vaccination used in the study, extra data for performing the cost-effectiveness analysis, and the supplementary results on sensitivity analyses. Table S1. The distribution of individuals by the level of sexual activity and age group. Table S2. Age distribution and mortality rate in Hong Kong. Table S3. Posterior distributions of inferred parameters. Table S4. Test performance parameters of cytology and HPV test. Table S5. Cost-effectiveness of guidelines-based screening strategies for unvaccinated cohorts. Table S6. ICERs of the most cost-effective screening strategy by vaccine uptake and duration of vaccine protection for unvaccinated cohorts. Table S7. Cost-effectiveness of screening strategies by annual discount rates for unvaccinated cohorts. Table S8. Sensitivity analysis on the estimated costs and health outcomes for unvaccinated cohorts by screen uptake. Table S9. Cost-effectiveness of guidelines-based screening strategies for vaccinated cohorts. Table S10. ICERs of the most cost-effective screening strategy by vaccine uptake and duration of vaccine protection for vaccinated cohorts. Table S11. Cost-effectiveness of screening strategies by annual discount rates for vaccinated cohorts. Table S12. Cost-effectiveness of screening strategies for vaccinated cohorts, with the assumption of faster HPV clearance rate and waning rate of natural immunity in males. Table S13. Sensitivity analysis of the estimated cost and health outcomes of screening strategies for vaccinated cohorts by screening uptake. Table S14. One-way sensitivity analysis on the cost-effectiveness of variants of strategy HPV + Genotyping for vaccinated cohorts. Table S15. Cost-effectiveness of variants of strategy HPV + Genotyping for vaccinated cohorts. Fig. S1. Schematic of the natural history model for high-risk HPV [file 12916_2023_2748_MOESM1_ESM.pdf]

Additional file 1: Supplementary Information

This file provides additional information on model description, parameterization and calibration, settings on cervical screening and HPV vaccination used in the study, extra data for performing the cost-effectiveness analysis, and the supplementary results on sensitivity analyses.

Content

Model description 2
Natural history 2
Sexual mixing 4
Adjusted contact rates 6
Model parameterization 7
HPV vaccination 12
Cervical screening 12
Cost-effectiveness analysis (CEA) 18
Cost parameters 18
Health outcomes 18
Cost-effectiveness analysis 18
Results for cost-effectiveness analysis 20

Model description

Natural history

Figure S1 shows the natural history of high-risk HPV (hrHPV) infection and cervical cancer among women in the model. The pathway is the same for each of the hrHPV classes considered in the study, namely HPV-16; HPV-18; HPV-OV ("other vaccine types"), which comprises the other five hrHPVs targeted by the 9vHPV vaccine, namely, HPV-31, 33, 45, 52, and 58; and HPV-NV (non-targeted types), which comprises all the non-vaccine hrHPVs (i.e., HPV-26, 35, 39, 51, 53, 56, 59, 66, 67, 68, 69, 73 and 82).[14] Individuals enter the population without HPV infection at birth and become sexually active as early as age 10. Individuals who are sexually active are susceptible to HPV infection. The force of infection for HPV infection depends on the prevalence of HPV infection in the opposite sex and the formation of sexual partnerships with the opposite sex at different ages and sexual activities, as well as the transmission probability (β_h) which is specific to HPV classes.[21, 24, 25] For women who are infected with HPV, the infection could progress to precancerous states (cervical intraepithelial neoplasia; CIN1, CIN2 and CIN3). The infection may also clear spontaneously in the health states HPV infection, CIN1 and CIN2. We assume that individuals with CIN3 would not recover naturally. Disease progression rates and clearance rates are assumed to depend on HPV type but not age.

Cervical cancer without symptoms may become symptomatic or progress to more advanced stages of cervical cancer without symptoms. In the absence of screening, cervical cancer is diagnosed only when symptoms develop, in which the patient will be treated accordingly. Women with cervical cancer diagnosed are subjected to the stage-specific probability of cancer-associated death.[26] The progression rates of cervical cancer and cervical cancer-related death rates are assumed to be independent of age, sexual activity level and HPV type.[21]

The basic compartmental epidemic models based on ordinary differential equations assume that the duration of each compartment is exponentially distributed.[27] For any given mean duration, the probability that the duration is shorter than the mean is higher in exponential distribution than in more biologically plausible distributions such as Erlang and lognormal distributions. Such a difference would have little effect on disease states milder than CIN3 because their durations are relatively short compared to cervical screening intervals. However, given that the expected duration of CIN3 is much longer (>10 years on average),[28] assuming that the duration of CIN3 is exponentially distributed might artificially lower the effectiveness of cervical screening. As such, we assume that the time from CIN3 to asymptomatic cervical cancer is an Erlang-4 distribution.[27, 29] This multiple-compartment component is important for the stochastic cohort simulation model which simulates the impact of cervical screening on the prevention of cervical cancer.

We refer to a local study for cervical cancer survival specified by the International Federation of Gynaecologists and Oncologists (FIGO) staging system.[30] The reported 5-year survival rates are 90.9%, 71.0%, 41.7% and 7.8% for FIGO stages I, II, III and IV, respectively. Cancer patients who remain alive 5 years after cancer diagnosis are regarded as cancer survivors.[31] In the model, we assume that cancer survivors will not be susceptible to new HPV infection because they may have their uterus and cervix removed with hysterectomy following cancer treatments.

We assume that transmission probability of HPV infection (β_h), HPV regression (τ_h^{HPV}) and waning of induced natural immunity (w_h) in males are the same as those in females.[21] We do not consider HPV-associated diseases among males.[21, 32]

Both dynamic and stochastic models involve the same natural history model, including state transition parameters, age structure, and sexual mixing. We also assume that individuals would experience age-related all-cause mortality that was observed in the general population.[33]

Figure S1. Schematic of the natural history model for high-risk HPV infection and cervical cancer among females.

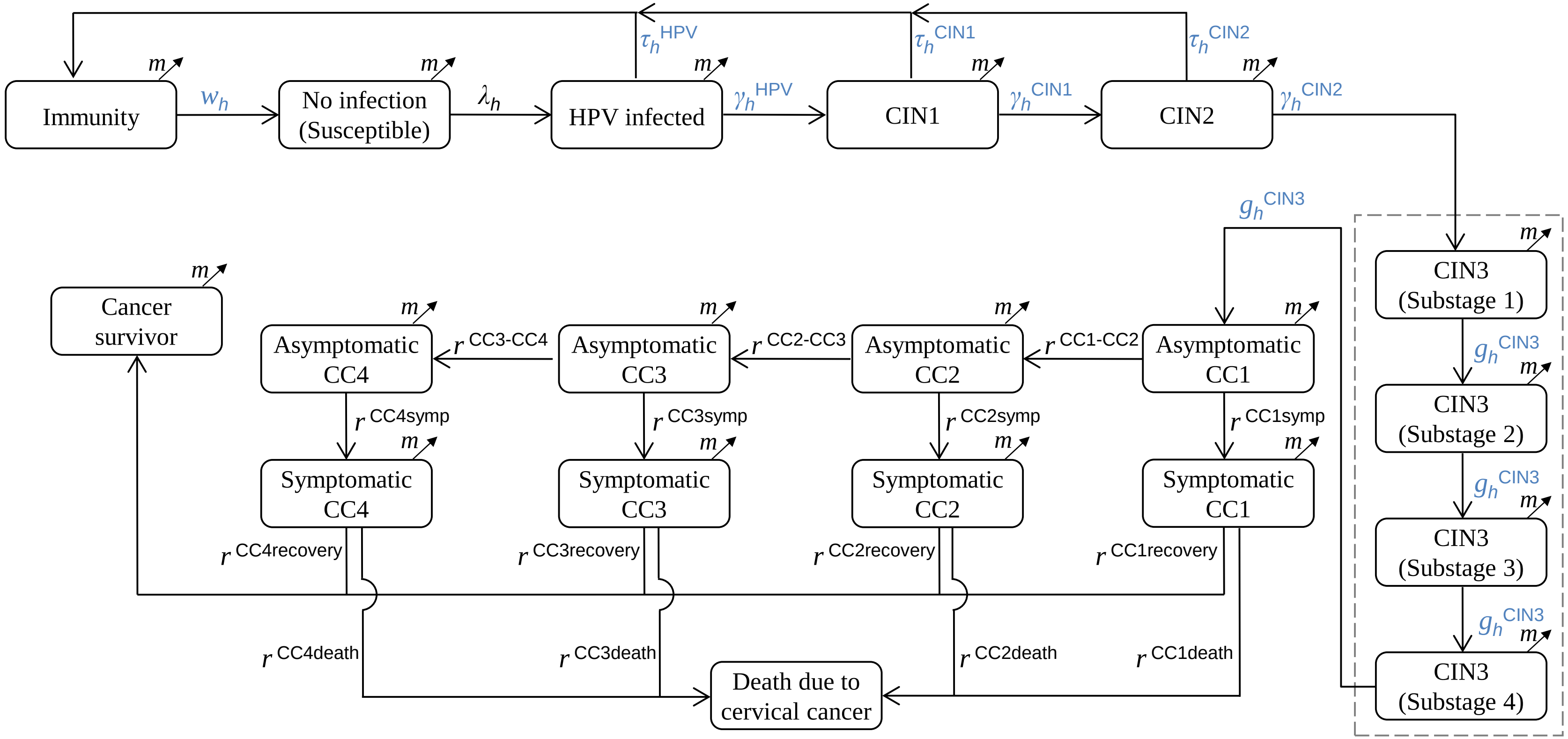

Abbreviations: CC1 to CC4, cervical cancer in International Federation of Gynecology and Obstetrics (FIGO) stage I to IV correspondingly; CIN1 to CIN3, cervical intraepithelial neoplasia grade 1 to 3.

λ_h is the force of infection (FOI) for HPV class h. γ_h^X and τ_h^X are the progression and clearance rates for disease state X with HPV class h. w_h is the waning rate of natural immunity against HPV class h. The progression of CIN3 to asymptomatic CC1 is assumed to follow an Erlang-4 distribution with mean $1/\gamma_h^{CIN3}$, i.e. $\gamma_h^{CIN3} = g_h^{CIN3} / 4$. An individual may transit to another health state or remain in the same health state. An individual may also experience an age-dependent all-cause mortality rate (m). Colored notations refer to the inferring parameters in model calibration, where λ_h is affected by the transmission probability (β_h). Other notations in black are based on the literature.

Sexual mixing

We use the survey data published by the Family Planning Association of Hong Kong (FPAHK) (http://www.famplan.org.hk) to construct the sexual activity matrix (Table S1) as follows:

1. Individuals aged below 10 or above 69 years are assumed to be sexually inactive.
2. The sexual activity distributions for individuals aged 13-14 years (text in red) are based on the data for Forms 1-2 students in The Report of Youth Sexuality Study 2011.
3. The sexual activity distributions for individuals aged 10-12 years (text in green) are linearly interpolated from the distributions in steps 1 and 2.
4. The sexual activity distributions for individuals aged 15-19 years (text in purple) are based on the data in Section Form 3- Form 7 in The Report of Youth Sexuality Study 2006.
5. The sexual activity distributions for individuals aged 20-24 years (text in magenta) are based on the data in Section Aged 18-27 Youths in The Report of Youth Sexuality Study 2006.
6. The sexual activity distributions for males aged 30-69 years (text in blue) are based on Table 7.5a in the 2001 Men's Health Survey.
7. The sexual activity distributions for females aged 30-69 years (text in orange) are extrapolated from the distribution for females aged 20-24 years (from step 5) assuming that the age effect on the distribution of low and high levels of sexual activity for females is the same as that for males (from step 6).
8. The data in both The Report of Youth Sexuality Study 2006 and Men's Health Survey 2001 suggest that those with a high level of sexual activity had an average of 2.5 sexual partners during the past 6 months.

Table S1. The distribution of individuals with no, low and high level of sexual activity in each age group.

	Sexual activity level (number of sexual partners during the 6 months)					
	Males			Females		
Age (years)	None (0)	Low (1)	High (>1)	None (0)	Low (1)	High (>1)
10-12	0.993	0.007	0.000	0.998	0.002	0.000
13-14	0.985	0.015	0.000	0.995	0.005	0.000
15-19	0.900	0.062	0.038	0.945	0.046	0.009
20-24	0.580	0.303	0.117	0.632	0.291	0.077
25-29	0.286	0.579	0.135	0.312	0.556	0.132
30-39	0.102	0.798	0.100	0.111	0.766	0.122
40-49	0.094	0.827	0.079	0.102	0.794	0.103
50-59	0.099	0.849	0.052	0.108	0.815	0.077
60-69	0.362	0.604	0.034	0.394	0.580	0.025

Note. Individuals with one and multiple sexual partners during the past 6 months are regarded as having low and high sexual activity levels, respectively.

Adjusted contact rates

For the transmission model to be internally consistent, the following balance rule must be satisfied at all times:

$$c_{f,a,u}\rho_{f,a,u,b,v}(t)N_{f,a,u}(t)=c_{m,b,v}\rho_{m,b,v,a,u}(t)N_{m,b,v}(t)$$

$$c_{f,a,u}\rho_{f,a,u,b,v}(t)N_{f,a,u}(t)=c_{m,b,v}\rho_{m,b,v,a,u}(t)N_{m,b,v}(t)$$

This balance rule simply states the fact that the number of sexual partnerships that females from stratum (*f*, *a*, *u*) form with males from stratum (*m*, *b*, *v*) is the same as the number of sexual partnerships that males from stratum (*m*, *b*, *v*) form with females from stratum (*f*, *a*, *u*). At any given time *t*, the degree to which the balance rule is violated could be measured by:

$$D_{a,u,b,v}(t)=\frac{c_{f,a,u}\rho_{f,a,u,b,v}(t)N_{f,a,u}(t)}{c_{m,b,v}\rho_{m,b,v,a,u}(t)N_{m,b,v}(t)}.$$

To ensure that the balance rule is satisfied at all times, the adjusted contact rates are:

$$c^{*}_{f,a,u,b,v}(t)=c_{f,a,u}\left(D_{a,u,b,v}(t)\right)^{-(1-\theta)}$$

$$c^{*}_{m,b,v,a,u}(t)=c_{m,b,v}\left(D_{a,u,b,v}(t)\right)^{\theta}$$

Following common practice, we choose $\theta = 0.5$ which means that the relevant parameters of females and males are adjusted to the same degree.[34]

Model parameterization

We estimate the model parameter values using the Markov chain Monte Carlo (MCMC) method and the Metropolis-Hasting algorithm with non-informative flat priors for all parameters (see Table S3).[35, 36] To calibrate the model, we consider the following fitting targets and the corresponding assumptions when formulating the likelihood function:

1. age-specific high-risk HPV prevalence in local studies,[14, 15] assuming that the prevalence follows binomial distribution;
2. proportion of HPV types in cervical cancer cases in the periods of 1972–73 and 1984–1986 from a local study,[37] assuming that the proportion follows binomial distribution;
3. age-specific cervical cancer incidence in the period of 1980–1984 based on the Hong Kong Cancer Registry statistics,[17] assuming that the incidence follows Poisson distributions;
4. disease progression and clearance (for different stages of HPV infection) from overseas clinical trials,[38, 39] assuming that the progression and clearance follow multinomial distributions.

We choose to fit the proportion of HPV types in cervical cancer cases and age-specific cancer incidence to data before the 1980s to minimize the confounding effect of screening on cervical cancer incidence (cervical screening was first introduced in clinical practice in Hong Kong in the 1980s).

Here we briefly describe the steps of parameter estimation based on the dynamic model.

1. We first construct hypothetical cohorts of females and males for the population. Without loss of generality, we set an overall population size of 100,000 for both genders. The gender- and age-specific distributions of the 1-year age groups (ages 1, 2, …, 84) and 85 or above are based on the figures from the Census & Statistics Department.[33] Table S2 shows the cohort size and mortality rate that aggregated for 5-year age groups of both sexes. We consider individuals aged 10 to 69 to be sexually active and stratify them into three sexual activity groups (see section *Sexual Mixing*).
2. We set the initial prevalence of the four HPV classes (HPV-16, HPV-18, HPV-OV, and HPV-NV) in sexually active age groups according to local studies.[14, 15] Given a parameter set of state transition rates and assortativeness variables, individuals will transit across different health states as described in the section *Natural history*. Upon ageing, individuals will move to the next older age group. For individuals before being sexually active (i.e., at ages 9 years or younger), we assume all of them are without HPV infection and that they will become susceptible to HPV infection when they become age 10. The force of infection (FOI) for HPV infection is age- and sex-specific and depends on the sexual mixing in forming sexual partnerships with the opposite sex. In the dynamic model, we assume that there is no cervical screening.
3. The proportion of individuals in different health states and age groups will change across time horizons and we trace the corresponding changes. The system is in an ‘equilibrium’ if the proportion of health states at the beginning of each year does not change (substantially) from the previous year. When the system reaches an equilibrium, we retrieve the simulated age-specific HPV prevalence of the four HPV classes and age-specific cervical cancer incidence at the populational level. We then compare the modelled outcomes with the empirical data.[14, 15, 17, 37-39] The given parameter set is considered to indicate a good fit (i.e., a high similarity) if the respective modelled outcomes do not statistically different from the empirical data.
4. The process is iterated by updating parameters that potentially show good calibration to the fitting targets via the Metropolis-Hasting algorithm.

Using this approach, we model the population-level HPV prevalence and cervical cancer incidence according to respective empirical data. This technique of parameter inference synthesizes the disease natural history model and multiple empirical targets without the need to observe all the transitions from individual-level data. This technique has also been used in international studies for building natural history models for cervical cancer screening and vaccination, and screening of colorectal and breast cancers.[22, 23, 40, 41]

Figure S2 illustrates the comparison between modelled and empirical fitting targets. The trace plot (Figure S3) and Geweke diagnostic [42] indicate that the MCMC chain converges. We estimate the posterior distribution by running the Metropolis-Hasting algorithm for 300,000 iterations with a burn-in of 150,000 iterations without thinning. Table S3 shows the summary statistics of the posterior distributions of the inferred parameters.

Table S2. Age distribution and mortality rate in Hong Kong.

	Female		Male	
Age (year)	Population size (2021)	Annual mortality rate, per 1,000 individuals (2021)	Population size (2021)	Annual mortality rate, per 1,000 individuals (2021)
1-4	93,322	0.17	98,352	0.15
5-9	140,635	0.08	148,811	0.07
10-14	139,688	0.10	149,710	0.12
15-19	130,018	0.13	134,882	0.22
20-24	161,479	0.15	164,735	0.35
25-29	243,900	0.21	214,507	0.38
30-34	299,040	0.24	227,988	0.49
35-39	353,241	0.30	234,502	0.71
40-44	351,589	0.60	235,022	1.16
45-49	340,807	1.10	242,182	2.00
50-54	330,158	1.68	245,084	3.14
55-59	345,976	2.47	284,806	4.80
60-64	313,580	3.59	300,222	7.28
65-69	252,678	5.24	239,557	11.59
70-74	188,822	8.52	181,929	18.96
75-79	100,476	16.92	96,666	32.77
80-84	85,475	32.46	79,701	50.47
85+	141,725	105.65	84,485	111.40

Source: Census and Statistics Department.[33]

Table S3. Posterior distributions of inferred parameters.

<table>
<tr><td colspan="2">(A) Inferred parameters on natural history</td><td colspan="4">Posterior median (95% credible interval [CrI])</td></tr>
<tr><td>Parameter</td><td>Description</td><td>HPV-16</td><td>HPV-18</td><td>HPV-OV</td><td>HPV-NV</td></tr>
<tr><td>β_h</td><td>Transmission probability per sexual partnership</td><td>0.75
(0.50, 0.96)</td><td>0.88
(0.60, 0.98)</td><td>0.93
(0.80, 0.99)</td><td>0.61
(0.50, 0.71)</td></tr>
<tr><td>$1/\gamma_h^{\mathrm{HPV}}$</td><td>Mean duration (yrs): progression from HPV infection to CIN1</td><td>8.7
(7.2, 11.3)</td><td>5.9
(4.3, 8.6)</td><td>10.7
(8.9, 12.8)</td><td>11.2
(9.4, 13.5)</td></tr>
<tr><td>$1/\gamma_h^{\mathrm{CIN1}}$</td><td>Mean duration (yrs): progression from CIN1 to CIN2</td><td>3.9
(2.7, 5.3)</td><td>3.4
(2.3, 5.2)</td><td colspan="2">2.7
(2.1, 3.9)</td></tr>
<tr><td>$1/\gamma_h^{\mathrm{CIN2}}$</td><td>Mean duration (yrs): progression from CIN2 to CIN3</td><td>4.2
(3.0, 6.4)</td><td>4.2
(2.9, 6.7)</td><td colspan="2">4.5
(2.9, 7.3)</td></tr>
<tr><td>$1/\gamma_h^{\mathrm{CIN3}}$</td><td>Mean duration (yrs): progression from CIN3 to CC1</td><td>22
(18, 28)</td><td>22
(16, 30)</td><td colspan="2">32
(20, 40)</td></tr>
<tr><td>$1/\tau_h^{\mathrm{HPV}}$</td><td>Mean duration (yrs): clearance of HPV infection</td><td>2.2
(1.9, 2.5)</td><td>1.4
(1.2, 1.7)</td><td>1.6
(1.5, 1.8)</td><td>1.6
(1.5, 1.7)</td></tr>
<tr><td>$1/\tau_h^{\mathrm{CIN1}}$</td><td>Mean duration (yrs): clearance of CIN1</td><td>3.2
(2.1, 4.8)</td><td>3.0
(2.2, 4.3)</td><td colspan="2">1.3
(1.1, 1.7)</td></tr>
<tr><td>$1/\tau_h^{\mathrm{CIN2}}$</td><td>Mean duration (yrs): clearance of CIN2</td><td>3.2
(2.5, 4.3)</td><td>3.2
(2.3, 4.4)</td><td colspan="2">1.7
(1.4, 2.4)</td></tr>
<tr><td>$1/w_h$</td><td>Mean duration (yrs) of natural immunity</td><td>15.9
(2.7, 83.2)</td><td>16.9
(3.6, 74.8)</td><td>0.68
(0.51, 1.69)</td><td>0
(assumed)</td></tr>
</table>

(B) Inferred parameters on sexual mixing		Posterior median (95% CrI)
W_1	Age (yrs) at which susceptibility and infectiousness begin to fall	21<br>(16, 25)
W_2	Age (yrs) at which susceptibility and infectiousness stop falling and begin to plateau	24<br>(21, 27)
μ	Relative transmission probability for individuals older than W_1	0.47<br>(0.41, 0.53)
ε_A	Degree of assortativeness for sexual mixing across ages	0.77<br>(0.29, 0.97)
ε_S	Degree of assortativeness for sexual mixing across sexual activity levels	0.98<br>(0.89, 0.99)
σ_g	Spread of age preference (yrs) in forming sexual partnership	2.05<br>(0.21, 4.90)

Adapted from Choi et al 2018.[20]
Abbreviations: CC1, cervical cancer stage I; CIN, cervical intraepithelial neoplasia; HPV-NV, non-targeted types; HPV-OV, other vaccine types that are targeted by the 9vHPV vaccines, i.e., HPV-31, 33, 45, 52, and 58; yrs, years.

Figure S2. Comparison of empirical data and the fitted model.

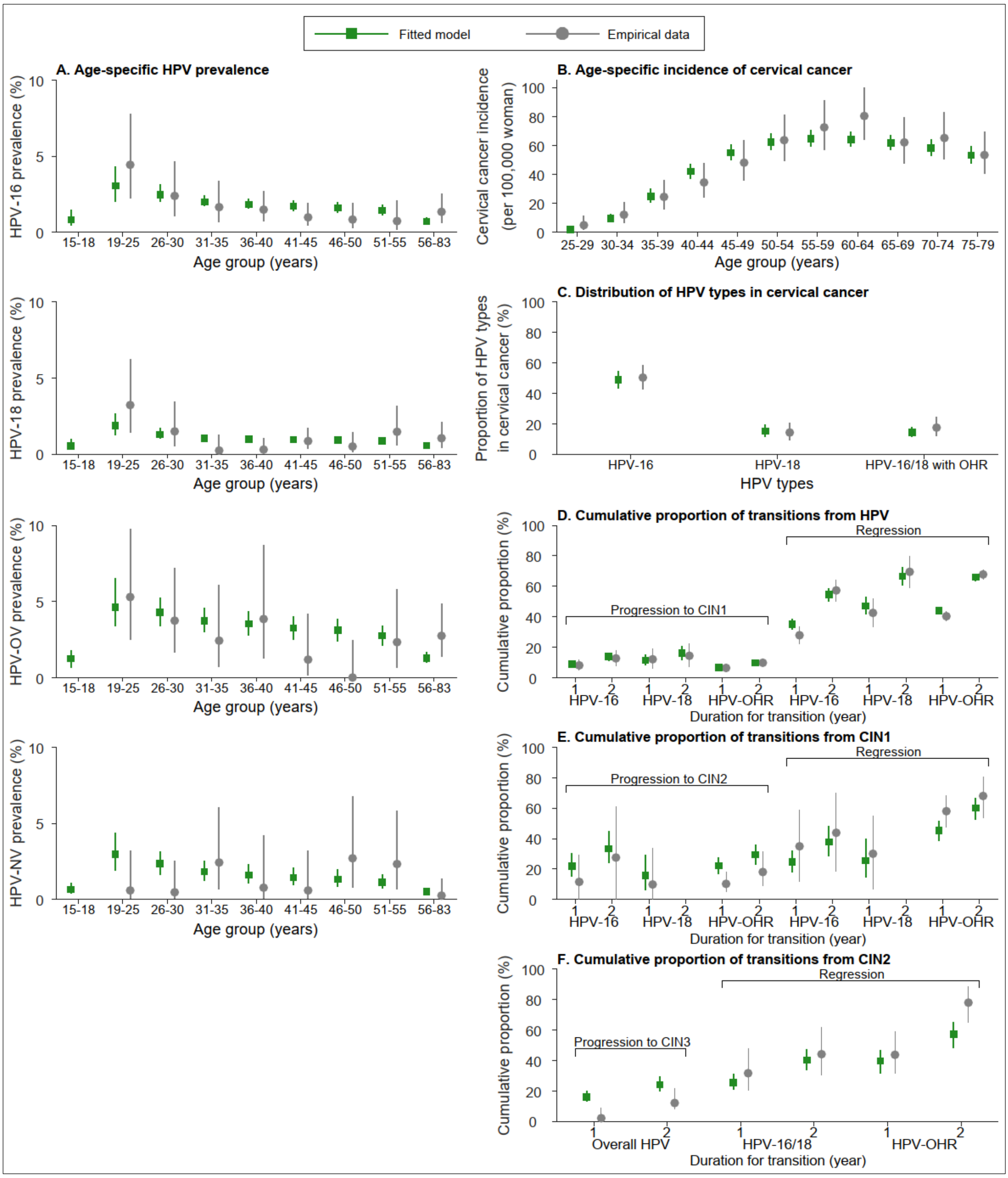

Adapted from Choi et al 2018.[20]

Abbreviations: CIN, cervical intraepithelial neoplasia; HPV-NV, non-targeted types; HPV-OHR, high-risk HPV types other than HPV-16/-18; HPV-OV, other vaccine types that are targeted by the 9vHPV vaccines, i.e., HPV-31, 33, 45, 52, and 58.

The gray circles and lines present the point estimates and the corresponding 95% confidence intervals of the empirical fitting targets. The green squares and lines present the median and the corresponding 95% percentile intervals based on the posterior samples of the fitted model.

Figure S3. Trace plots and the posterior distributions of the fitted parameters.

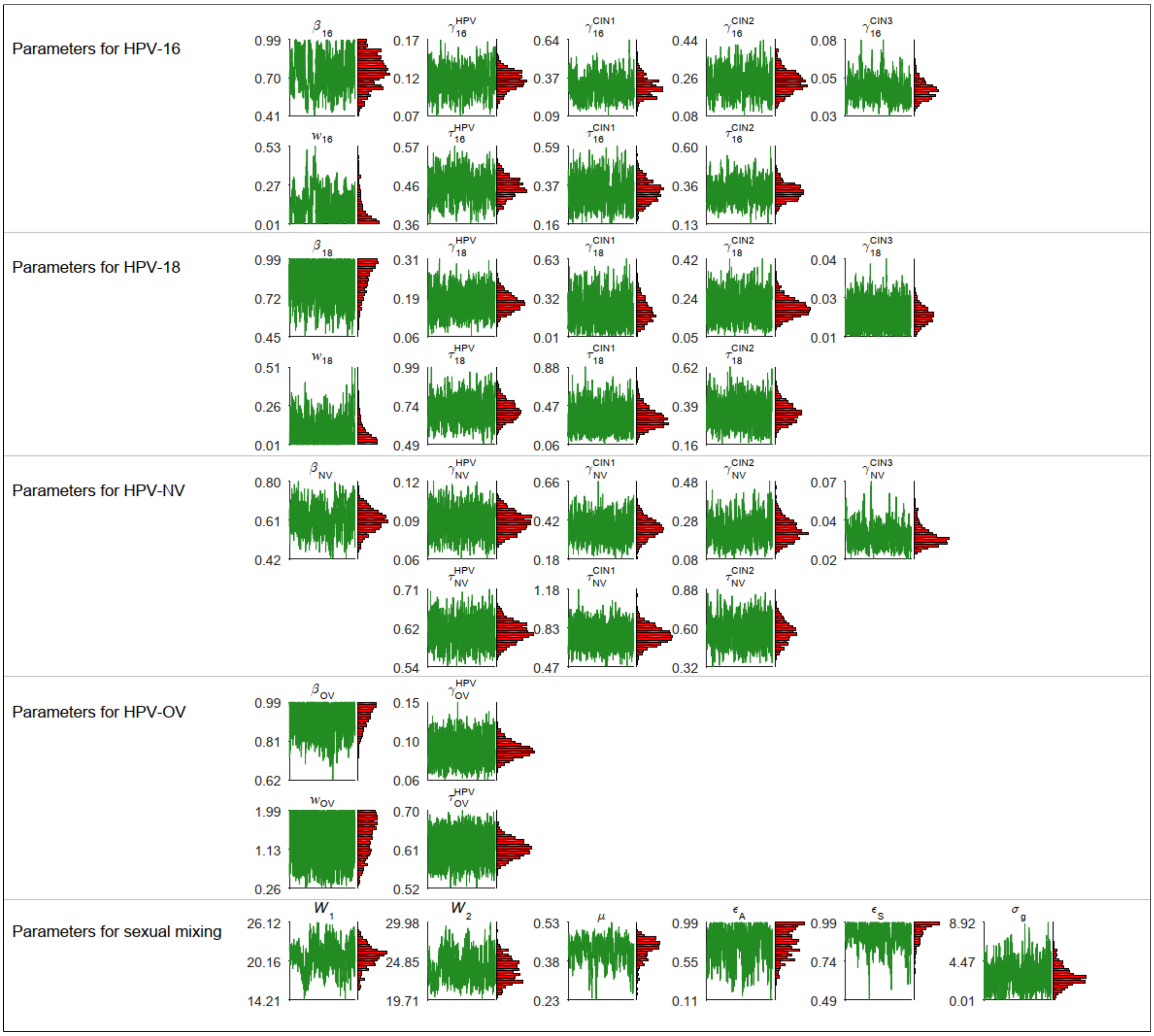

Adapted from Choi et al 2018.[20]
Abbreviations: CIN, cervical intraepithelial neoplasia; HPV-NV, non-targeted types; HPV-OV, other vaccine types that are targeted by the 9vHPV vaccines, i.e., HPV-31, 33, 45, 52, and 58.
Note. Refer to Table S3 for the definition of the parameters.

HPV vaccination

In Hong Kong, routine HPV vaccination was recently included in the childhood immunization program in 2019.[47] Schoolgirls who are in primary five and six (equivalent to age 11–12 years) are eligible to receive 9vHPV without charges. The latest statistics indicated that 86% of the primary six schoolgirls had received two doses of 9vHPV vaccines via the immunization program as of December 2021.[48] Before the implementation of routine HPV vaccination, females could get vaccinated when the first-generation HPV (bivalent [2vHPV] or quadrivalent [4vHPV]) vaccines were available on the market in 2008. We assume that the opportunistic vaccine uptake among females aged 12 years increased from 2.4% in 2008 to 12% in 2016 according to local surveys,[49, 50] and remained steady at 12% till 2019 when routine vaccination commenced.[43] We also assume that females received the 2vHPV / 4vHPV vaccines via opportunistic vaccination before the routine HPV vaccination commenced in 2019 and that all schoolgirls who wish to get vaccinated would only receive the 9vHPV vaccines via the immunization program in and after 2019. We consider that the routine HPV vaccination program would continue regardless of cancer incidence in the future.

In the model, we adopt the vaccine-induced efficacies of the 9vHPV vaccines for vaccine-targeted hrHPV classes from the corresponding clinical trials and local HPV epidemiology.[3, 14, 46] Specifically, the vaccine efficacy of the 9vHPV vaccines against HPV-16, HPV-18, and HPV-OV was 95.5% (95% confidence interval [CI] = 90.0%, 98.4%), 95.8% (84.1%, 99.5%), and 96.0% (94.4%, 97.2%), respectively. Furthermore, we assume a constant degree of protection and a fixed duration for non-lifelong duration (i.e., 30- and 20-year) scenarios. We also assume that the vaccines do not provide type-specific protection if an individual has been infected with the respective HPV type before vaccination.

Cervical screening

The Cervical Screening Programme (CSP) in Hong Kong was launched in 2004.[18] CSP recommended eligible women aged 25–64 years to be screened annually for the first two years, with a 3-year regular screening interval if the results of the first two yearly screens remain negative. Approximately 70% of eligible women indicated in surveys that they had been screened at least once.[18] In mid-2021, the CSP indicated the use of the HPV DNA test (HPV test) as a primary or co-test for cervical screenings for women aged 30–64 years, in addition to cytology-based screening.[51] HPV DNA testing for high-risk HPV types can be incorporated into cervical screening by being used as a triage for atypical squamous cells of undetermined significance (ASCUS) after cytology, as a co-testing with cytology, or as a primary stand-alone tool. We refer to the management guidelines proposed by the Hong Kong College of Obstetricians and Gynaecologists (HKCOG) for the follow-up actions per various combinations of cytology and HPV DNA test results.[19] The guidelines recommend using HPV testing for women aged 30 years or above. Following the guidelines, screening would start at the age of 25 years with cytology as the primary for all screening strategies examined. Under primary HPV test and co-test as primary (i.e., strategies B1-B3 and C1-C3 in Figure S4, respectively), the primary screening would switch to HPV test and co-test at age 30 years, respectively. For co-test strategies, HPV tests would still be performed among women aged 25–29 years as a co-test when they have abnormal cytology. When evaluating the impacts of screening strategies, we assume that alternative strategies will be adopted starting in 2022. For older age cohorts that have already started cervical screening, we assume that they would follow the strategy A1 Cytology-only before 2022 and would switch to the alternative strategies afterward.

For women who would undergo screening in or after the current calendar year, we assume that (i) 70% of women aged 25–64 years would attend screening when they reach 25 years old, based on the statistics of local surveys;[52, 53] and (ii) all women in (i) who have initiated screening would fully comply the recommended or proposed screening intervals of the screening strategies. We assume full compliance for evaluating the maximum potential impact of screening strategies. Regarding the screening pattern before the current calendar year, we assume that (i) 70% screening uptake among eligible women aged 25–64 years after the commencement of CSP; (ii) among (i), 75% would comply with regular screening, i.e., attending the 3-year screening of cytology-based screening following the initial screening guidelines; and (iii) the rest of (ii) would attend a longer screening interval at 6 years upon receiving consecutive test results.[52, 53] For the time before the commencement of CSP, we assume that (i) 40% screening uptake among eligible women aged 25–64 years;[52, 54, 55] (ii) 60% of (i) would comply with a regular screening at 1-, 2-, or 3-year intervals;[52] and (iii) the rest of (ii) will attend the screening at a longer interval of 44 months upon receiving negative test results.[52] Regarding the relationship between screening preference and individuals' vaccination status, we assume that screening uptake would be the same among vaccinated and unvaccinated individuals.

Furthermore, currently, the electronic health record systems of immunization and screening in Hong Kong are not linked.[18, 56] Joining electronic platforms is voluntary and requires users to initiate registration and

consent for sharing corresponding information between the public and private sectors. Alternatively, parents could keep a paper card of immunization for their children. That is, individual-level vaccination characteristics (e.g., age at vaccination and number of doses received) may rely on recall, or the immunization record card if it is still available, when women attend cervical screenings nearly 15–20 years after they were vaccinated in their adolescence. As such, we assume a single screening strategy for both vaccinated and unvaccinated women in the vaccinated cohorts.

We adopt the findings reported in the meta-analyses for the test performance of cytology and HPV testing (Table S4).[57-61] If screening suggested high-grade lesions, a colposcopic-directed biopsy would be done. Colposcopic directed biopsy is generally considered the reference for diagnosis and therefore we assume that colposcopic directed biopsy to be 100% accurate in the model. We also assume that liquid-based cytology (LBC) instead of conventional cytology would be used, given the advantages that LBC allows the performance of reflex HPV testing and that LBC reduces the rate of unsatisfactory cytology sampling.[19]

Figure S4. Schematic presentation of evaluated cervical screening algorithms that are currently indicated in Hong Kong.

(a) Primary cytology with a 3-yearly routine screening interval, with two managements for ASCUS cytology test results

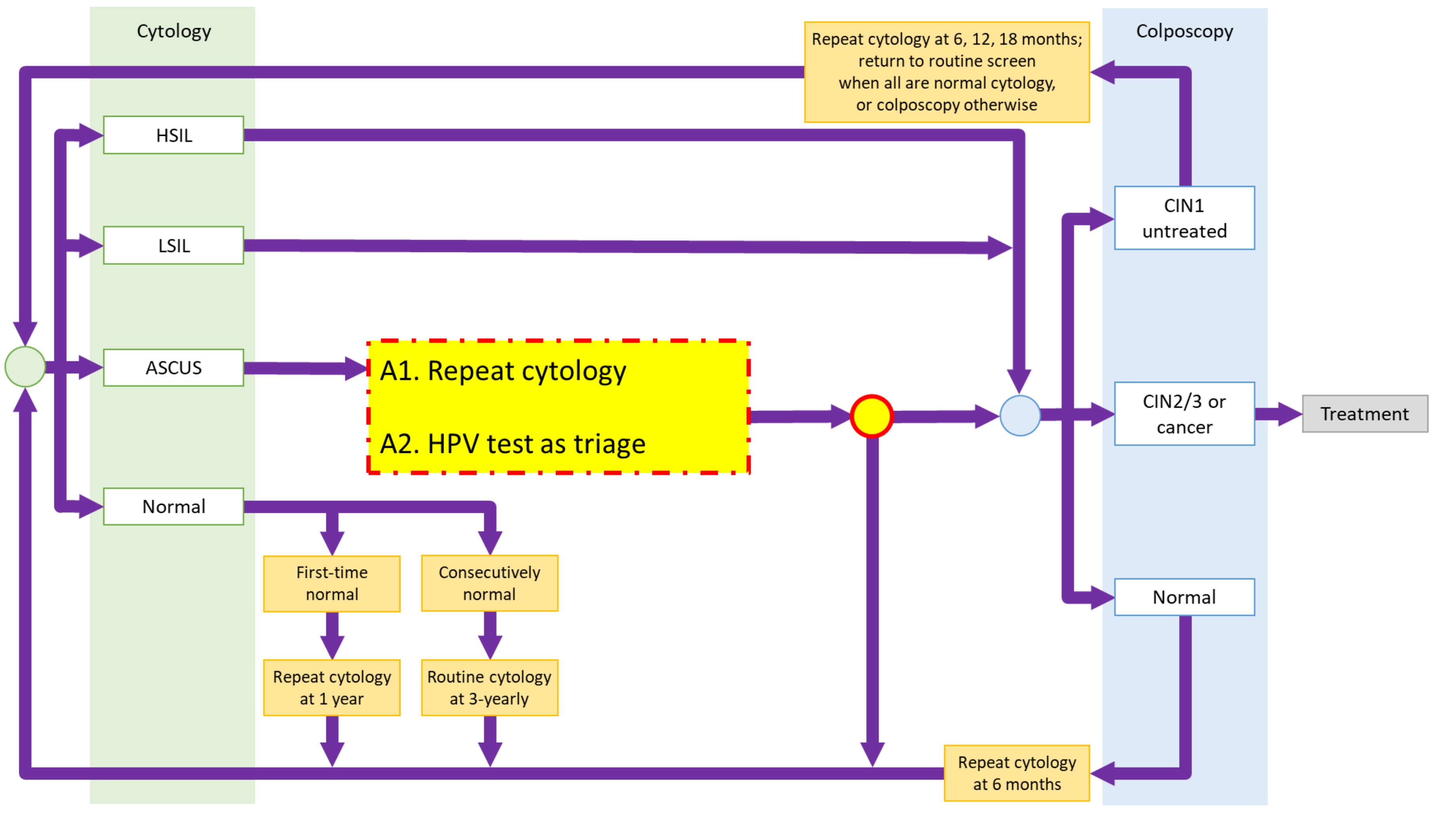

(b) Primary HPV test with a 5-yealry routine screening interval, with three managements for HPV test-positive cases

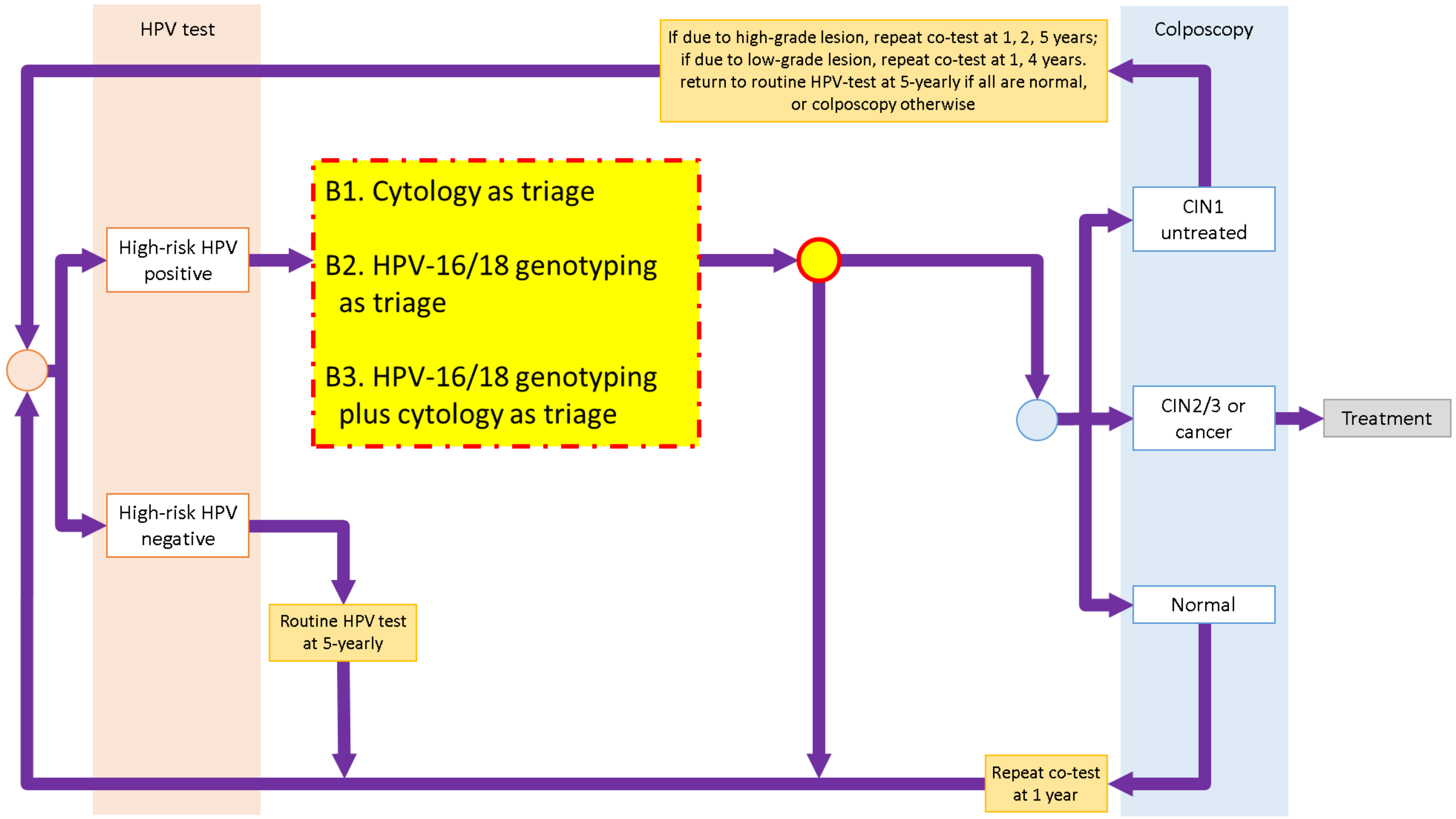

(c) Co-test of cytology and HPV test as primary with a 5-yealry routine screening interval, with three managements for normal cytology and HPV test-positive cases

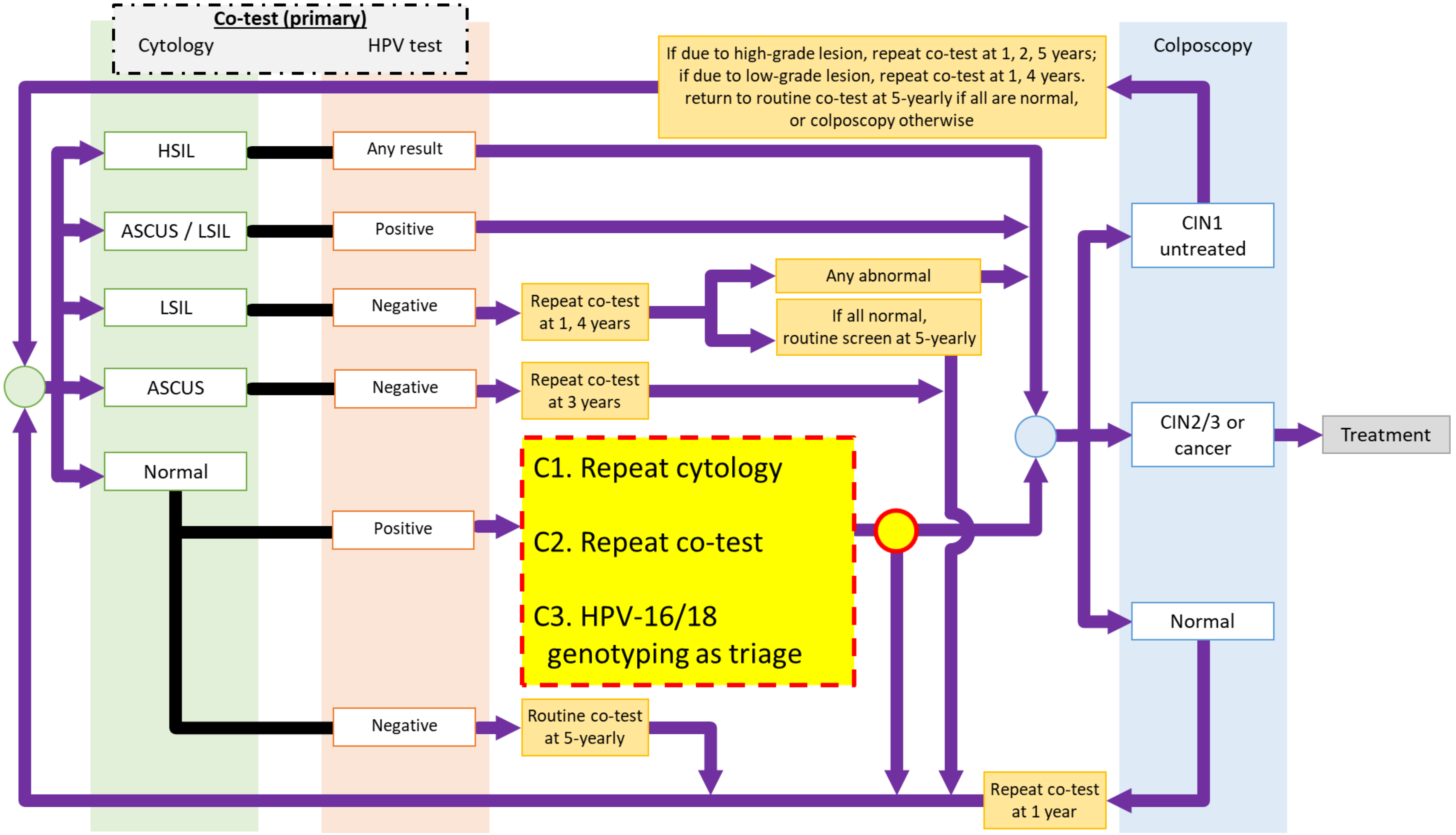

1

Table S4. Test performance parameters of cytology and HPV test.

Performance	Distribution	References
Liquid-based cytology		
Sensitivity		
CIN1	*T*(0.659, 0.719, 0.690)	[57]
CIN2/3	*T*(0.666, 0.827, 0.755)	[58]
Cervical cancer	*T*(0.95, 1.00, 1.00)	Assumed
Specificity	*T*(0.944, 0.985, 0.971)	[57]
HPV DNA test		
Sensitivity		
CIN1	*T*(0.763, 0.843, 0.806)	[59]
CIN2/3	*T*(0.896, 0.953, 0.926)	[58]
Cervical cancer	*T*(0.95, 1.00, 1.00)	Assumed
Specificity	*T*(0.868, 0.941, 0.911)	[59]
Probability of returning HPV-16/18 genotype test result given		
HPV-16/18 infected	*T*(0.9, 1.0, 0.95)	Assumed [60]
other high-risk HPVs (HPV-OV/NV) infected	*T*(0.0, 0.2, 0.1)	Assumed [61]
no high-risk HPV infected	*T*(0.0, 0.2, 0.1)	Assumed [61]

T(*a*, *b*, *c*) denotes a triangular distribution that ranges from *a* to *b* with mode *c*.

Cost-effectiveness analysis (CEA)

Cost parameters

We estimate the costs for screening and treatments based on the private charges in the public healthcare system (listed in the Gazette[62, 69]) which is led by the Hospital Authority and Department of Health and accounts for approximately 90% of hospitalization in Hong Kong.[63] We assume that public hospitals make these charges just for covering their costs instead of making profits. We also assume that the private charges do not take into account the factors, such as hospital construction, land premium or investment returns, which are considered by healthcare service providers in the private sector. The private charges are comparable to or lower than the "public charges for non-eligible persons" which have been made on a cost-recovery basis (both charges are offered by public hospitals by the Hospital Authority).[70, 71] Therefore, we assume that the private charges are close to the actual costs. While charges and costs are not exactly equivalent, such charges are the most publicly available relevant data in Hong Kong at the moment and have been used in many local health care CEAs.[20, 72] The use of charges listed in the Gazette has also been adopted for estimating the direct medical costs for Chinese colorectal cancer Chinese patients in a local cost analysis.[73] Furthermore, we also refer to the fee of Pap smear and HPV testing charged in local private clinics or health centers. The underlying assumptions are provided below.

The costs of screening comprise the following:

1. The cost of liquid-based cytology;
2. The cost of HPV testing for routine screening;
3. The cost of colposcopy and biopsy for the follow-up of a low-grade squamous intraepithelial lesion (LSIL) and high-grade squamous intraepithelial lesion (HSIL) per corresponding management of screening strategies.
4. Time and transportation costs for women to go to clinics for screening, where the time cost is based on the average income of women by age group.[66, 67]

The procedures of cancer treatment per the stage at diagnosis comprise the following:

1. Stage I: Wertheim's hysterectomy;
2. Stage II/III: radiotherapy (planning and teletherapy), chemotherapy (1 line of therapy), and brachytherapy;
3. Stage IV: radiotherapy (planning and teletherapy) and chemotherapy (2 lines of therapy).

We estimate the treatment costs for CIN2/3 and cervical cancer based on private charges for standard treatments in public hospitals. We include women's time and transportation costs in the overall aggregated treatment costs for CIN2/3 and cervical cancer. The private charges for inpatients include the fees for general nursing, core pathology investigations, catering and also domestic services.[71] We assume that the costs of treating CIN2 and CIN3 are the same. In Hong Kong, the standard treatment procedure for CIN2/3 is excision and ablative treatment is not suitable. The treatment cost of cervical cancer comprises the cost of hospitalization for receiving Wertheim's hysterectomy, brachytherapy, and overnight infusion chemotherapy. The costs of cancer treatment are different by the stage at diagnosis (Table 1 in the main text). We assume that 67.4% of the women who die from cervical cancer would receive palliative care with a mean hospitalization duration of 42.5 days.[74] Furthermore, upon positive HPV DNA test results, genotyping for HPV-16/18 may be used as a triage strategy.[19] No extra cost of HPV genotyping is accumulated because HPV genotyping is performed simultaneously with the initial HPV test in each round of screening as a usual clinical practice.

Health outcomes

It has been highlighted that the weighting of health-related quality of life (HRQoL) for different health states regarding cervical screening may be divergent across different studies.[6, 75] We use life year (LY) and quality-adjusted life year (QALY) as the metrics to quantify the health outcomes of screening strategies, while we consider LY as the primary metric because it is more commonly used in international CEAs on cervical screening. When calculating QALYs, we use health utility parameters from internationally published cervical screening cost-effectiveness studies because analogous data are not available in Hong Kong. We adopt the health utilities based on the values used in the CEA conducted by Bains et al (2019) who reviewed and summarized the health utilities reported in the literature.[6] In our analysis, QALY is estimated by summing women's health utilities incurred throughout their lifetimes. Health utility is reduced when (i) attending cervical screening visits; (ii) upon receiving abnormal screening results; (iii) upon having diagnosis and treatment (if any) of CIN; and (iv) during treatment of cervical cancer. We assume that there is no health utility loss for undiagnosed or asymptomatic cervical lesions and cervical cancers.[25] Furthermore, we assume that cancer survivors have lower health utilities for their first 5 years after treatment.[31]

Cost-effectiveness analysis

To assess the long-term impact of HPV vaccination and cervical screening, we compute the costs and health outcomes associated with cervical cancer over the lifetime of women in each cohort. The age structure and sexual mixing in the stochastic model and the dynamic model are the same. We set the discount rate at 3% annually for both costs and health outcomes and the outcomes were discounted starting from the year 2022. As a sensitivity analysis, we considered annual discount rates at 0% and 6%, and screening participation rates at 50% and 100%. To account for parameter uncertainty, we conduct probabilistic sensitivity analysis (PSA) which includes (i) 100 parameter sets relating to disease epidemiology (e.g., the natural history of HPV transmission, vaccine efficacy and test performance) and (ii) 100 parameter sets on costs and health utilities. A total of 10,000 combinations of parameters are sampled using Latin hypercube sampling. We consider the incremental cost-effectiveness ratio (ICER), which is defined as the incremental cost divided by the incremental health outcome, when comparing two strategies. That is, $ICER = \frac{C_2 - C_1}{E_2 - E_1}$, where C_i and E_i are the mean cost and mean health outcomes of strategy i that are estimated from PSA, respectively. In general, for an increase in health outcomes (i.e., $E_2 - E_1 > 0$), strategy 2 is deemed cost-effective if the corresponding ICER is below the willingness to pay (WTP) threshold. When ordering screening strategies, the strategy that has the lowest average cost-effectiveness ratio against the situation of no screening will be ranked first, and then followed by strategies that return the lowest ICERs with the previous non-dominating strategies.[66] We consider a strategy strongly dominated when it is less effective but more costly than an alternative strategy, and a strategy extendedly (weakly) dominated when it generates a higher ICER than a more effective strategy. There is no consensus on the WTP threshold in Hong Kong. The World Health Organization (WHO) no longer recommends directly using 1-3 times the gross domestic product per capita (GDPpc) as the WTP threshold.[77] Therefore, we refer to the WTP threshold used in local CEAs which informed policymaking. Some recent local studies used the threshold of US$50,000 per QALY gained,[78, 79] while some used 1 to 3 GDPpc for each QALY gained.[20, 80, 81] In this study, we use a relatively conservative WTP threshold of 1 GDPpc per unit of health outcomes (LYs or QALYs) as the WTP threshold. Because GDPpc highly depends on the economic environment in the region (e.g., economic activities might be affected during the COVID-19 pandemic), we consider an average of GDPpc over the last five years instead of the most recent year. The average GDPpc was US$47,792 / HK$372,778 (ranging US$45,855-49,629) in Hong Kong during 2017-2021.[76]

We perform one-way sensitivity analysis on selected strategy comparisons. We follow the approach used by Demarteau et al and Jit et al to estimate the contributions (coefficients) of parameters to the outcomes using linear regression models.[82, 83] Based on the PSA, we include health economics and test performance parameters as independent variables in the linear regression models with the differences in cost, LY and QALY as the dependent variables separately. Given the fitted linear regression models, we multiply the end points of the 95% confidence interval of the parameters (based on respective distributions in Table 1 of the main text) by the model coefficients to get the predicted outcomes for varying the parameters. We then obtain the corresponding estimated ICERs by dividing the predicted difference in cost by the predicted difference in health outcomes (LY / QALY).

We conduct a scenario to assess the impact of assuming the same HPV natural history parameters in both genders. Similar to the settings in other HPV modeling studies, we assume that parameters that are related to HPV infection were the same in both genders.[21, 24, 32] Some clinical studies observed that the durations of HPV infection and natural immunity upon recovery from HPV infection were shorter in males than that in females.[84-86] For example, the median duration of HPV-16 and HPV-18 infection among males from the HIM study was 12.2 and 7.3 months, respectively;[84] the corresponding median times among females was 7.3 and 6.9 months based on the Ludwig-McGill cohort study, respectively.[85] Some clinical findings indicated that the time to clearance of any-HPV infection may be shorter (i.e., a faster clearance) in males.[84, 87] As a scenario analysis, we assume that the HPV clearance rate (τ_h^{HPV}) and waning rate of natural immunity (w_h) in males are 2 times faster than that in females for all HPV classes. That is, $\tau^{M}_{h}{}^{\text{HPV}}$ (males) = $2 \times \tau^{F}_{h}{}^{\text{HPV}}$ (females), and w^{M}_{h} (males) = $2 \times w^{F}_{h}$ (females). With this assumption, we re-generate parameter sets that are calibrated to empirical data including HPV prevalence and cervical cancer incidence (see section *Model parameterization*) and then estimate the resulting costs, LYs and QALYs of the screening strategies accordingly.

Results for cost-effectiveness analysis

Results for unvaccinated cohorts.

Table S5 presents the cost-effectiveness table of the guidelines-based screening strategies for unvaccinated cohorts when the screening uptake was 70%.

Table S6 presents the ICERs of the most cost-effective (i.e., having the lowest ICER) guidelines-based screening strategies (with the recommended routine screening intervals) when compared with no screening across scenarios of vaccine uptake and duration of vaccine protection for the unvaccinated cohorts. The corresponding ICERs varied only slightly (<2%) across scenarios of vaccine uptake and duration of vaccine protection among unvaccinated cohorts.

Table S7 presents the cost-effectiveness of the guidelines-based screening strategies in the scenarios of an annual discount rate at 0% (i.e., undiscounted) and 6%. When the annual discount rate was 0%, the estimated ICERs decreased when compared to the scenario of a 3% annual discount rate in the base case. The estimated ICERs increased when the annual discount rate was 6%.

Figure S5 presents the one-way sensitivity analysis for comparing strategy A2 (Cytology + HPV reflex; i.e., using HPV test to triage cytology result of ASCUS) vs A1 (Cytology-only) when LY was the metric for health outcomes. The figure also presents the findings for comparing A1 vs No screening when QALY was the metric for health outcomes.

Table S8 presents the findings when the screening participation rate was 50% and 100%. The CEAs and the estimated ICERs were not affected by the screening participation rates under the assumption that women who have initiated screening would follow recommended subsequent screening visits and necessary clinical appointments.

Results for vaccinated cohorts.

Table S9 presents the cost-effectiveness table of the guidelines-based screening strategies for cohorts with the routine vaccination program (vaccinated cohorts) when 9vHPV vaccines provided lifelong protection, the vaccine uptake was 85%, and the screening uptake was 70%.

Table S10 presents the ICERs of the most cost-effective (i.e., having the lowest ICER) guidelines-based screening strategies (with the recommended routine screening intervals) when compared with no screening across scenarios of vaccine uptake and duration of vaccine protection for the vaccinated cohorts.

Table S11 presents the cost-effectiveness of the guidelines-based screening strategies in the scenarios of an annual discount rate at 0% (i.e., undiscounted) and 6%. When the annual discount rate was 0%, the estimated ICERs decreased when compared to the scenario of a 3% annual discount rate in the base case. Strategy B2 (HPV + Genotyping) became cost-effective under the WTP threshold at 1 GDPpc when compared with no screening. The estimated ICERs increased when the annual discount rate was 6%.

Figure S6 presents the one-way sensitivity analysis for comparing strategies B2 (HPV + Genotyping) and A1 (Cytology-only) vs No screening when LY and QALY were the metrics for health outcomes, respectively.

Table S12 presents the incremental CEA for the scenario analysis under the assumption that the HPV clearance rate and waning rate of natural immunity in males were 2 times faster than that in females for all HPV classes. The findings are based on the PSA with parameter sets that are calibrated to empirical data under this assumption. When the vaccine uptake is 85% and the vaccine provides lifelong protection, the comparative cost-effectiveness is comparable to the original setting in which the natural history parameters of HPV infection are assumed the same in both genders. For example, the ICERs of the first non-dominated strategy (compared with no screening) increased by less than 2% when compared to the original setting.

Table S13 presents the findings when the screening participation rate was 50% and 100%. The CEAs and the estimated ICERs were not affected by the screening participation rates under the assumption that women who have initiated screening would follow recommended subsequent screening visits and necessary clinical appointments.

Table S14 presents the one-way sensitivity analysis of the cost-effectiveness when applying de-intensification approaches in strategy B2 with the initial guidelines-based recommendation, under the base-case assumptions of 85% vaccine uptake and lifelong vaccine protection and 70% screening uptake.

Table S15 presents the cost-effectiveness table of the de-intensified variants and the guidelines-based strategy B2 (HPV + Genotyping) for cohorts with the routine vaccination program (vaccinated cohorts) when 9vHPV vaccines provided lifelong protection, the vaccine uptake was 85%, and screening uptake was 70%.

Table S5. Cost-effectiveness of guidelines-based screening strategies for cohorts without a routine vaccination program (unvaccinated cohorts) when the screening uptake was 70%.

	Total cost, LY and QALY Mean (95% percentile interval)			Difference compared with strategy A1 (Cyto-only) Mean (95% percentile interval)			Incremental CEA (compared with the previous non-dominated strategy)	
Screening strategies	Total cost [a] (US$'M)	Total LY [a]	Total QALY [a]	Total cost [a] (US$'M)	Total LY [a]	Total QALY [a]	ICER per LY (US$) [b]	ICER per QALY (US$) [b]
B2 (HPV + Geno)	7.7 (6.3, 9.1)	241,711 (241,676, 241,740)	241,639 (241,590, 241,685)	-0.3 (-1.6, 1.1)	-5.6 (-9.0, -1.7)	-27.2 (-45.3, -11.6)	21,644	ExtDom
A1 (Cyto-only)	8.0 (6.4, 9.4)	241,717 (241,685, 241,745)	241,667 (241,625, 241,703)	-	-	-	ExtDom	23,389
A2 (Cyto + Reflex HPV)	8.0 (6.4, 9.5)	241,719 (241,688, 241,747)	241,667 (241,625, 241,704)	0.05 (-0.006, 0.12)	2.4 (1.5, 3.6)	0.3 (-1.4, 2.0)	40,137	181,297
B1 (HPV + Cyto)	8.0 (6.6, 9.4)	241,715 (241,681, 241,743)	241,642 (241,593, 241,687)	0.1 (-1.2, 1.4)	-1.8 (-4.2, 1.5)	-24.8 (-42.1, -9.4)	Dom	Dom
B3 (HPV + Geno and Cyto)	8.2 (6.7, 9.7)	241,716 (241,682, 241,744)	241,640 (241,590, 241,686)	0.3 (-1.1, 1.7)	-1.0 (-3.5, 2.5)	-27.0 (-46.6, -10.1)	Dom	Dom
C1 (Co-test + Repeat cyto)	9.2 (7.4, 10.6)	241,721 (241,690, 241,748)	241,650 (241,605, 241,690)	1.2 (0.4, 1.9)	4.5 (2.8, 6.9)	-16.9 (-28.7, -4.3)	528,110	Dom
C2 (Co-test + Repeat co-test)	9.6 (7.8, 11.2)	241,722 (241,691, 241,748)	241,634 (241,583, 241,682)	1.6 (0.6, 2.7)	5.1 (2.7, 8.5)	-32.2 (-52.0, -11.3)	ExtDom	Dom
C3 (Co-test + Geno)	9.9 (8.0, 11.5)	241,722 (241,692, 241,749)	241,632 (241,579, 241,682)	1.9 (0.9, 3.1)	5.9 (3.4, 9.4)	-34.6 (-56.8, -12.0)	538,180	Dom

Abbreviations: Cyto: cytology; Dom, dominated; ExtDom, extendedly dominated; Geno, HPV-16/18 genotyping; LY, life year; QALY, quality-adjusted life year.
Notes. [a] Total cost, LY, and QALY, and corresponding differences compared with A1 (cytology-only) are presented per 10,000 individuals. Means and 95% percentile intervals are obtained based on simulation. [b] ICERs are expressed as the incremental mean cost divided by the incremental mean LY or mean QALY correspondingly, compared with the previous non-dominated strategy. The ICERs of the first non-dominated strategy are compared with no screening. ICERs that are below the WTP threshold at 1 GDPpc (US$47,792) are underlined and highlighted. A strategy is dominated (Dom) if it has higher costs and worse outcomes than an alternative strategy. A strategy is extendedly dominated (ExtDom) if the ICER for the strategy is higher than that of the next more effective, non-dominated alternative strategy. [d] Assuming that the vaccine uptake was 85% among women via the routine immunization program and that the HPV vaccines provided lifelong protection.

Table S6. Incremental cost-effectiveness ratios (ICERs) of the most cost-effective guidelines-based screening strategy across scenarios of vaccine uptake and duration of vaccine protection for cohorts without the routine vaccination program (unvaccinated cohorts).

(a) Life years (LYs) as metric for health outcomes					
ICERs per LY gained (US$)		Vaccine uptake among the age cohorts via a routine immunization program [a]			
Vaccine protection	Strategy	85%	75%	50%	25%
Lifelong	B2 (HPV + Genotyping) vs No screening	21,644	21,639	21,629	21,620
30-year	B2 (HPV + Genotyping) vs No screening	21,641	21,638	21,627	21,617
20-year	B2 (HPV + Genotyping) vs No screening	21,616	21,613	21,596	21,584
(b) Quality-adjusted life years (QALYs) as metric for health outcomes					
ICERs per QALY gained (US$)		Vaccine uptake among the age cohorts via a routine immunization program			
Vaccine protection	Strategy	85%	75%	50%	25%
Lifelong	A1 (Cytology-only) vs No screening	23,389	23,346	23,242	23,149
30-year	A1 (Cytology-only) vs No screening	23,375	23,332	23,227	23,131
20-year	A1 (Cytology-only) vs No screening	23,332	23,290	23,179	23,082

Note. [a] All ICERs are below the WTP threshold at 1 GDPpc (US$47,792).

Table S7. Cost-effectiveness of guidelines-based screening strategies at (a) 0% (i.e., undiscounted) and (b) 6% annual discount rates for cohorts without a routine vaccination program (unvaccinated cohorts) when the screening uptake was 70%.

(a) 0% annual discount rate	Total cost, LY and QALY Mean (95% percentile interval)			Incremental CEA (compared with the previous non-dominated strategy)	
Screening strategies	Total cost [a] (US$'M)	Total LY [a]	Total QALY [a]	ICER per LY (US$) [b]	ICER per QALY (US$) [b]
B2 (HPV + Geno)	11.6 (9.5, 13.7)	435,847 (435,767, 435,909)	435,738 (435,639, 435,828)	9,093	ExtDom
A1 (Cyto-only)	11.9 (9.5, 14.1)	435,857 (435,784, 435,919)	435,780 (435,692, 435,856)	ExtDom	9,378
A2 (Cyto + Reflex HPV)	12.0 (9.6, 14.1)	435,861 (435,789, 435,922)	435,782 (435,694, 435,858)	28,495	54,584
B1 (HPV + Cyto)	12.1 (9.9, 14.3)	435,854 (435,777, 435,915)	435,743 (435,645, 435,832)	Dom	Dom
B3 (HPV + Geno and Cyto)	12.4 (10.1, 14.7)	435,855 (435,779, 435,916)	435,740 (435,641, 435,830)	Dom	Dom
C1 (Co-test + Repeat cyto)	13.8 (11.2, 15.9)	435,866 (435,795, 435,925)	435,758 (435,668, 435,839)	383,051	Dom
C2 (Co-test + Repeat co-test)	14.5 (11.8, 16.9)	435,867 (435,797, 435,925)	435,736 (435,637, 435,826)	ExtDom	Dom
C3 (Co-test + Geno)	14.9 (12.1, 17.4)	435,869 (435,799, 435,926)	435,733 (435,633, 435,825)	387,849	Dom
(b) 6% annual discount rate					
Screening strategies	Total cost [a] (US$'M)	Total LY [a]	Total QALY [a]	ICER per LY (US$) [b]	ICER per QALY (US$) [b]
B2 (HPV + Geno)	5.7 (4.7, 6.7)	158,515 (158,497, 158,530)	158,461 (158,431, 158,490)	44,183	ExtDom
A1 (Cyto-only)	5.9 (4.7, 7.0)	158,518 (158,501, 158,533)	158,481 (158,457, 158,502)	ExtDom	52,973
A2 (Cyto + Reflex HPV)	6.0 (4.8, 7.1)	158,519 (158,503, 158,534)	158,480 (158,456, 158,502)	Dom	Dom
B1 (HPV + Cyto)	6.0 (4.9, 7.0)	158,517 (158,500, 158,532)	158,463 (158,433, 158,491)	52,393	Dom
B3 (HPV + Geno and Cyto)	6.1 (5.0, 7.2)	158,517 (158,500, 158,532)	158,461 (158,430, 158,490)	Dom	Dom
C1 (Co-test + Repeat cyto)	6.8 (5.5, 7.9)	158,520 (158,505, 158,535)	158,467 (158,440, 158,492)	ExtDom	Dom
C2 (Co-test + Repeat co-test)	7.1 (5.8, 8.3)	158,521 (158,505, 158,535)	158,455 (158,423, 158,487)	ExtDom	Dom
C3 (Co-test + Geno)	7.3 (6.0, 8.6)	158,521 (158,506, 158,535)	158,454 (158,419, 158,486)	718,345	Dom

Abbreviations: Cyto: cytology; Dom, dominated; ExtDom, extendedly dominated; Geno, HPV-16/18 genotyping; LY, life year; QALY, quality-adjusted life year.
Notes. [a] Total cost, LY, and QALY are presented per 10,000 individuals. Means and 95% percentile intervals are obtained based on simulation. [b] ICERs are expressed as the incremental mean cost divided by the incremental mean LY or mean QALY correspondingly, compared with the previous non-dominated strategy. The ICERs of the first non-dominated strategy are compared with no screening. ICERs that are below the WTP threshold at 1 GDPpc (US$47,792) are underlined and highlighted. A strategy is dominated (Dom) if it has higher costs and worse outcomes than an alternative strategy. A strategy is extendedly dominated (ExtDom) if the ICER for the strategy is higher than that of the next more effective, non-dominated alternative strategy.

Figure S5. One-way sensitivity analysis of estimated ICER for comparing (a) A2 (Cytology + HPV reflex) vs A1 (Cytology-only) when using LY as the metric for health outcomes and (b) A1 (Cytology-only) vs No screening when using QALY as the metric for health outcomes for cohorts without the routine vaccination program (unvaccinated cohorts).

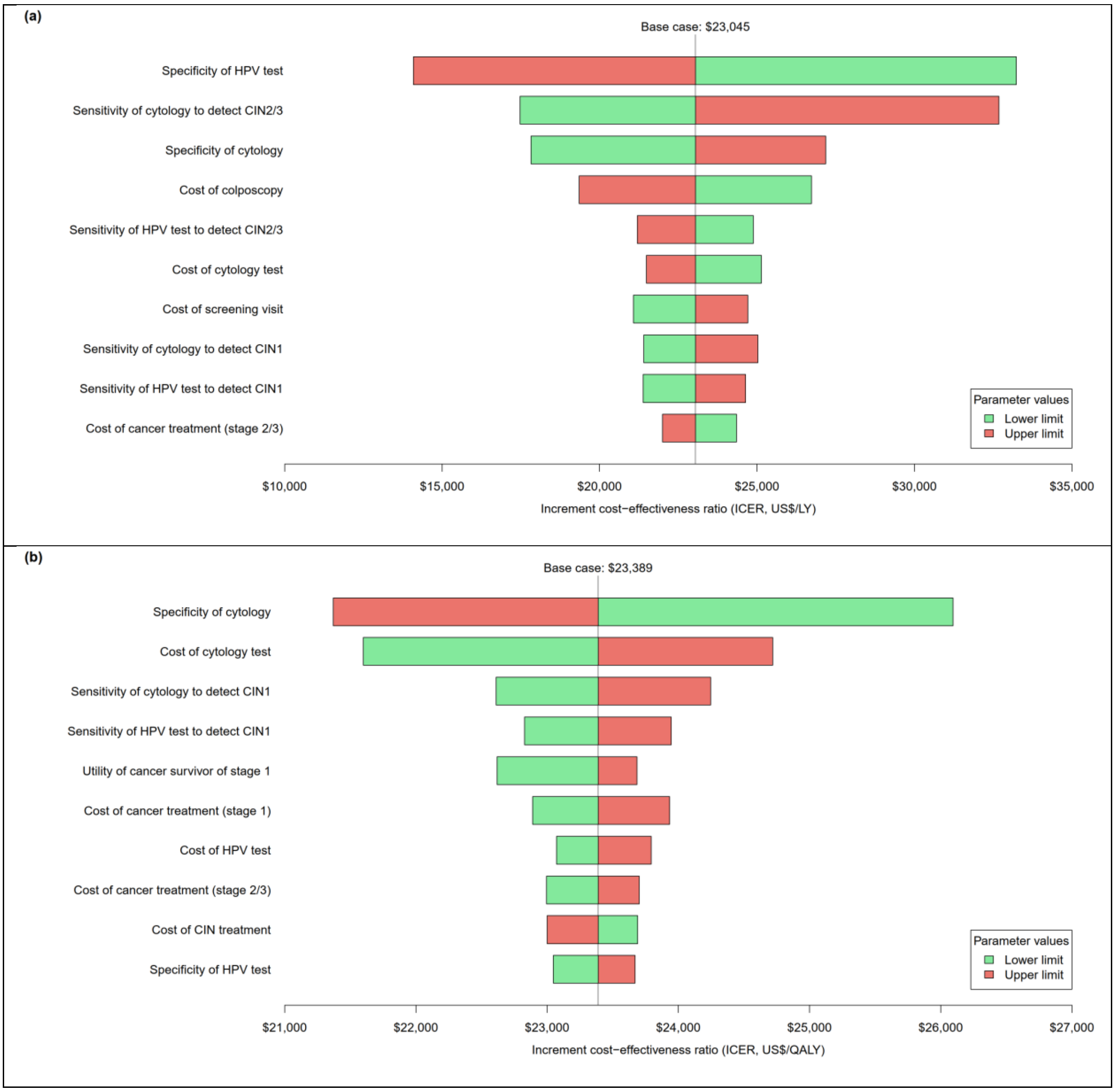

Abbreviations: CIN, cervical intraepithelial neoplasia; LY, life year; QALY, quality-adjusted life year.

Table S8. Sensitivity analysis on the estimated costs and health outcomes of guidelines-based screening strategies for cohorts without a routine vaccination program (unvaccinated cohorts) when the screening uptake was (a) 50% and (b) 100%.

(a) Screening uptake at 50%	Total cost, LY and QALY<br>Mean (95% percentile interval)			Difference compared with strategy A1 (Cyto-only)<br>Mean (95% percentile interval)			Incremental CEA (compared with the previous non-dominated strategy)	
Screening strategies	Total cost [a] (US$'M)	Total LY [a]	Total QALY [a]	Total cost [a] (US$'M)	Total LY [a]	Total QALY [a]	ICER per LY (US$) [b]	ICER per QALY (US$) [b]
B2 (HPV + Geno)	6.2<br>(5.2, 7.3)	241,643<br>(241,598, 241,681)	241,643<br>(241,598, 241,681)	-0.2<br>(-1.2, 0.8)	-4.01<br>(-6.40, -1.21)	-19.4<br>(-32.3, -8.3)	21,644	ExtDom
A1 (Cyto-only)	6.4<br>(5.2, 7.5)	241,647<br>(241,605, 241,684)	241,647<br>(241,605, 241,684)	-	-	-	ExtDom	23,389
A2 (Cyto + Reflex HPV)	6.5<br>(5.3, 7.5)	241,649<br>(241,607, 241,685)	241,649<br>(241,607, 241,685)	0.04<br>(-0.004, 0.09)	1.69<br>(1.07, 2.60)	0.2<br>(-1.0, 1.4)	40,137	181,297
B1 (HPV + Cyto)	6.5<br>(5.3, 7.5)	241,646<br>(241,602, 241,683)	241,646<br>(241,602, 241,683)	0.1<br>(-0.9, 1.0)	-1.27<br>(-3.02, 1.08)	-17.7<br>(-30.1, -6.7)	Dom	Dom
B3 (HPV + Geno and Cyto)	6.6<br>(5.5, 7.7)	241,647<br>(241,603, 241,684)	241,647<br>(241,603, 241,684)	0.2<br>(-0.8, 1.2)	-0.69<br>(-2.48, 1.80)	-19.3<br>(-33.3, -7.2)	Dom	Dom
C1 (Co-test + Repeat cyto)	7.3<br>(6.0, 8.3)	241,651<br>(241,609, 241,686)	241,651<br>(241,609, 241,686)	0.9<br>(0.3, 1.4)	3.25<br>(1.97, 4.94)	-12.0<br>(-20.5, -3.1)	528,110	Dom
C2 (Co-test + Repeat co-test)	7.6<br>(6.3, 8.8)	241,651<br>(241,609, 241,687)	241,651<br>(241,609, 241,687)	1.2<br>(0.5, 2.0)	3.64<br>(1.96, 6.08)	-23.0<br>(-37.2, -8.1)	ExtDom	Dom
C3 (Co-test + Geno)	7.8<br>(6.4, 9.0)	241,652<br>(241,610, 241,687)	241,652<br>(241,610, 241,687)	1.4<br>(0.6, 2.2)	4.19<br>(2.40, 6.73)	-24.7<br>(-40.6, -8.6)	538,180	Dom

(b) Screening uptake at 100%	Total cost, LY and QALY<br>Mean (95% percentile interval)			Difference compared with strategy A1 (Cyto-only)<br>Mean (95% percentile interval)			Incremental CEA (compared with the previous non-dominated strategy)	
Screening strategies	Total cost [a] (US$'M)	Total LY [a]	Total QALY [a]	Total cost [a] (US$'M)	Total LY [a]	Total QALY [a]	ICER per LY (US$) [b]	ICER per QALY (US$) [b]
B2 (HPV + Geno)	9.9<br>(8.0, 11.8)	241,812<br>(241,792, 241,830)	241,726<br>(241,680, 241,770)	-0.4<br>(-2.3, 1.5)	-8.02<br>(-12.80, -2.42)	-38.9<br>(-64.7, -16.6)	21,644	ExtDom
A1 (Cyto-only)	10.3<br>(8.1, 12.3)	241,820<br>(241,802, 241,837)	241,765<br>(241,734, 241,793)	-	-	-	ExtDom	23,389
A2 (Cyto + Reflex HPV)	10.3<br>(8.1, 12.4)	241,824<br>(241,806, 241,840)	241,766<br>(241,733, 241,795)	0.08<br>(-0.009, 0.17)	3.39<br>(2.15, 5.21)	0.4<br>(-2.0, 2.9)	40,137	181,297
B1 (HPV + Cyto)	10.4<br>(8.4, 12.3)	241,818<br>(241,799, 241,834)	241,730<br>(241,684, 241,773)	0.1<br>(-1.8, 1.9)	-2.54<br>(-6.05, 2.17)	-35.4<br>(-60.2, -13.4)	Dom	Dom
B3 (HPV + Geno and Cyto)	10.7<br>(8.6, 12.8)	241,819<br>(241,801, 241,835)	241,727<br>(241,678, 241,773)	0.4<br>(-1.5, 2.4)	-1.37<br>(-4.96, 3.60)	-38.6<br>(-66.6, -14.4)	Dom	Dom
C1 (Co-test + Repeat cyto)	12.0<br>(9.6, 13.9)	241,827<br>(241,811, 241,841)	241,741<br>(241,702, 241,780)	1.7<br>(0.6, 2.8)	6.50<br>(3.93, 9.88)	-24.1<br>(-41.0, -6.2)	528,110	Dom
C2 (Co-test + Repeat co-test)	12.6<br>(10.2, 14.9)	241,828<br>(241,812, 241,842)	241,719<br>(241,667, 241,770)	2.4<br>(0.9, 3.9)	7.28<br>(3.93, 12.16)	-46.0<br>(-74.3, -16.2)	ExtDom	Dom
C3 (Co-test + Geno)	13.0<br>(10.5, 15.3)	241,829<br>(241,813, 241,842)	241,716<br>(241,661, 241,770)	2.7<br>(1.2, 4.4)	8.38<br>(4.80, 13.45)	-49.4<br>(-81.2, -17.2)	538,180	Dom

Abbreviations: Cyto: cytology; Dom, dominated; ExtDom, extendedly dominated; Geno, HPV-16/18 genotyping; LY, life year; QALY, quality-adjusted life year.
Notes. [a] Total cost, LY, and QALY, and corresponding differences compared with A1 (cytology-only) are presented per 10,000 individuals. Means and 95% percentile intervals are obtained based on simulation. [b] ICERs are expressed as the incremental mean cost divided by the incremental mean LY or mean QALY correspondingly, compared with the previous non-dominated strategy. The ICERs of the first non-dominated strategy are compared with no screening. ICERs that are below the WTP threshold at 1 GDPpc (US$47,792) are underlined and highlighted. A strategy is dominated (Dom) if it has higher costs and worse outcomes than an alternative strategy. A strategy is extendedly dominated (ExtDom) if the ICER for the strategy is higher than that of the next more effective, non-dominated alternative strategy. [d] Assuming that the vaccine uptake was 85% among women via the routine immunization program and the HPV vaccines provided lifelong protection.

Table S9. Cost-effectiveness of guidelines-based screening strategies for cohorts with the routine vaccination program (vaccinated cohorts) when 9vHPV vaccines provided lifelong protection, the vaccine uptake was 85%, and the screening uptake was 70%.

	Total cost, LY and QALY<br>Mean (95% percentile interval)			Difference compared with strategy A1 (Cyto-only)<br>Mean (95% percentile interval)			Incremental CEA (compared with the previous non-dominated strategy)	
Screening strategies	Total cost [a] (US$'M)	Total LY [a]	Total QALY [a]	Total cost [a] (US$'M)	Total LY [a]	Total QALY [a]	ICER per LY (US$) [b]	ICER per QALY (US$) [b]
B2 (HPV + Geno)	8.0<br>(6.8, 9.1)	297,129<br>(297,116, 297,136)	297,096<br>(297,077, 297,112)	-0.6<br>(-1.7, 0.3)	0.04<br>(-0.32, 0.36)	-8.7<br>(-18.8, -0.7)	59,863	ExtDom
B1 (HPV + Cyto)	8.3<br>(7.0, 9.4)	297,129<br>(297,116, 297,136)	297,095<br>(297,076, 297,111)	-0.4<br>(-1.4, 0.5)	0.18<br>(-0.05, 0.57)	-9.5<br>(-19.5, -1.4)	1,846,885	ExtDom
B3 (HPV + Geno and Cyto)	8.4<br>(7.1, 9.6)	297,129<br>(297,117, 297,136)	297,094<br>(297,073, 297,111)	-0.3<br>(-1.3, 0.7)	0.18<br>(-0.05, 0.56)	-10.9<br>(-21.7, -2.4)	ExtDom	ExtDom
A1 (Cyto-only)	8.7<br>(7.1, 10.1)	297,128<br>(297,116, 297,136)	297,105<br>(297,089, 297,117)	-	-	-	Dom	78,003
A2 (Cyto + Reflex HPV)	8.7<br>(7.1, 10.1)	297,129<br>(297,117, 297,136)	297,104<br>(297,087, 297,116)	0.022<br>(-0.051, 0.085)	0.18<br>(-0.05, 0.50)	-1.0<br>(-2.1, -0.1)	Dom	Dom
C1 (Co-test + Repeat cyto)	9.4<br>(7.8, 10.6)	297,129<br>(297,117, 297,136)	297,086<br>(297,064, 297,105)	0.7<br>(0.03, 1.3)	0.26<br>(-0.06, 0.64)	-18.8<br>(-30.0, -7.4)	ExtDom	Dom
C2 (Co-test + Repeat co-test)	9.5<br>(7.9, 10.8)	297,129<br>(297,117, 297,136)	297,080<br>(297,055, 297,103)	0.8<br>(0.022, 1.6)	0.32<br>(0.0009, 0.78)	-24.6<br>(-38.1, -10.3)	8,589,436	Dom
C3 (Co-test + Geno)	9.6<br>(8.0, 11.0)	297,129<br>(297,117, 297,136)	297,079<br>(297,053, 297,102)	0.9<br>(0.1, 1.8)	0.32<br>(0.0009, 0.78)	-26.1<br>(-40.7, -11.0)	Dom	Dom

Abbreviations: Cyto: cytology; Dom, dominated; ExtDom, extendedly dominated; Geno, HPV-16/18 genotyping; LY, life year; QALY, quality-adjusted life year.
Notes. [a] Total cost, LY, and QALY, and corresponding differences compared with A1 (cytology-only) are presented per 10,000 individuals. Means and 95% percentile intervals are obtained based on simulation. [b] ICERs are expressed as the incremental mean cost divided by the incremental mean LY or mean QALY correspondingly, compared with the previous non-dominated strategy. The ICERs of the first non-dominated strategy are compared with no screening. ICERs that are below the WTP threshold at 1 GDPpc (US$47,792) are underlined. A strategy is dominated (Dom) if it has higher costs and worse outcomes than an alternative strategy. A strategy is extendedly dominated (ExtDom) if the ICER for the strategy is higher than that of the next more effective, non-dominated alternative strategy.

Table S10. Incremental cost-effectiveness ratios (ICERs) of the most cost-effective guidelines-based screening strategy across scenarios of vaccine uptake and duration of vaccine protection for cohorts in the routine vaccination program (vaccinated cohorts) when the screening uptake was 70%.

(a) Life years (LYs) as metric for health outcomes					
ICERs per LY gained (US$)		Vaccine uptake among the age cohorts via a routine immunization program [a]			
Vaccine protection	Strategy	85%	75%	50%	25%
Lifelong	B2 (HPV + Genotyping) vs No screening	59,836	49,218	32,600	23,581
30-year	B2 (HPV + Genotyping) vs No screening	57,008	47,301	32,039	23,491
20-year	B2 (HPV + Genotyping) vs No screening	48,509	41,511	29,851	22,849
(b) Quality-adjusted life years (QALYs) as metric for health outcomes					
ICERs per QALY gained (US$)		Vaccine uptake among the age cohorts via a routine immunization program [a]			
Vaccine protection	Strategy	85%	75%	50%	25%
Lifelong	A1 (Cytology-only) vs No screening	78,003	61,994	38,458	26,289
30-year	A1 (Cytology-only) vs No screening	72,943	58,506	37,177	25,921
20-year	A1 (Cytology-only) vs No screening	59,322	49,409	33,772	24,884

Note. [a] ICERs that are below the WTP threshold at 1 GDPpc (US$47,792) are underlined and highlighted.

Table S11. Cost-effectiveness of guidelines-based screening strategies at (a) 0% (i.e., undiscounted) and (b) 6% annual discount rates for cohorts with the routine vaccination program (vaccinated cohorts) when 9vHPV vaccines provided lifelong protection, the vaccine uptake was 85%, and the screening uptake was 70%.

(a) 0% annual discount rate	Total cost, LY and QALY Mean (95% percentile interval)			Incremental CEA (compared with the previous non-dominated strategy)	
Screening strategies	Total cost [a] (US$'M)	Total LY [a]	Total QALY [a]	ICER per LY (US$) [b]	ICER per QALY (US$) [b]
B2 (HPV + Geno)	17.3 (14.4, 20.0)	744,101 (744,041, 744,143)	744,017 (743,943, 744,075)	24,464	26,686
B1 (HPV + Cyto)	18.0 (14.9, 20.9)	744,102 (744,042, 744,143)	744,015 (743,941, 744,073)	1,029,235	Dom
B3 (HPV + Geno and Cyto)	18.3 (15.2, 21.3)	744,102 (744,042, 744,143)	744,011 (743,936, 744,071)	ExtDom	Dom
A1 (Cyto-only)	19.1 (15.3, 22.6)	744,101 (744,041, 744,142)	744,041 (743,974, 744,090)	Dom	74,335
A2 (Cyto + Reflex HPV)	19.1 (15.4, 22.6)	744,102 (744,043, 744,143)	744,039 (743,972, 744,089)	Dom	Dom
C1 (Co-test + Repeat cyto)	21.0 (17.2, 24.2)	744,102 (744,043, 744,144)	743,996 (743,922, 744,056)	ExtDom	Dom
C2 (Co-test + Repeat co-test)	21.4 (17.5, 24.7)	744,103 (744,044, 744,144)	743,980 (743,899, 744,048)	4,935,225	Dom
C3 (Co-test + Geno)	21.8 (17.8, 25.3)	744,103 (744,044, 744,144)	743,976 (743,893, 744,047)	Dom	Dom
(b) 6% annual discount rate					
Screening strategies	Total cost [a] (US$'M)	Total LY [a]	Total QALY [a]	ICER per LY (US$) [b]	ICER per QALY (US$) [b]
B2 (HPV + Geno)	4.6 (4.0, 5.1)	165,659 (165,656, 165,660)	165,644 (165,637, 165,650)	138,150	ExtDom
B1 (HPV + Cyto)	4.7 (4.1, 5.3)	165,659 (165,656, 165,660)	165,644 (165,636, 165,650)	3,118,685	Dom
B3 (HPV + Geno and Cyto)	4.8 (4.1, 5.3)	165,659 (165,656, 165,660)	165,643 (165,635, 165,650)	ExtDom	Dom
A1 (Cyto-only)	4.8 (4.1, 5.5)	165,659 (165,656, 165,660)	165,647 (165,641, 165,652)	Dom	252,623
A2 (Cyto + Reflex HPV)	4.9 (4.1, 5.5)	165,659 (165,656, 165,660)	165,647 (165,641, 165,652)	ExtDom	Dom
C1 (Co-test + Repeat cyto)	5.1 (4.4, 5.7)	165,659 (165,656, 165,660)	165,638 (165,629, 165,647)	ExtDom	Dom
C2 (Co-test + Repeat co-test)	5.2 (4.4, 5.8)	165,659 (165,656, 165,660)	165,636 (165,625, 165,646)	13,146,318	Dom
C3 (Co-test + Geno)	5.2 (4.5, 5.9)	165,659 (165,656, 165,660)	165,635 (165,624, 165,646)	Dom	Dom

Abbreviations: Cyto: cytology; Dom, dominated; ExtDom, extendedly dominated; Geno, HPV-16/18 genotyping; LY, life year; QALY, quality-adjusted life year.

Notes. [a] Total cost, LY, and QALY are presented per 10,000 individuals. Means and 95% percentile intervals are obtained based on simulation. [b] ICERs are expressed as the incremental mean cost divided by the incremental mean LY or mean QALY correspondingly, compared with the previous non-dominated strategy. The ICERs of the first non-dominated strategy are compared with no screening. ICERs that are below the WTP threshold at 1 GDPpc (US$47,792) are underlined and highlighted. A strategy is dominated (Dom) if it has higher costs and worse outcomes than an alternative strategy. A strategy is extendedly dominated (ExtDom) if the ICER for the strategy is higher than that of the next more effective, non-dominated alternative strategy.

Figure S6. One-way sensitivity analysis of estimated ICER for comparing (a) B2 (HPV + Genotyping) vs No screening when using LY as metric for health outcomes and (b) A1 (Cytology-only) vs No screening when using QALY as metric for health outcomes for cohorts with the routine vaccination program (vaccinated cohorts) when 9vHPV vaccines provided lifelong protection, the vaccine uptake was 85%, and the screening uptake was 70%.

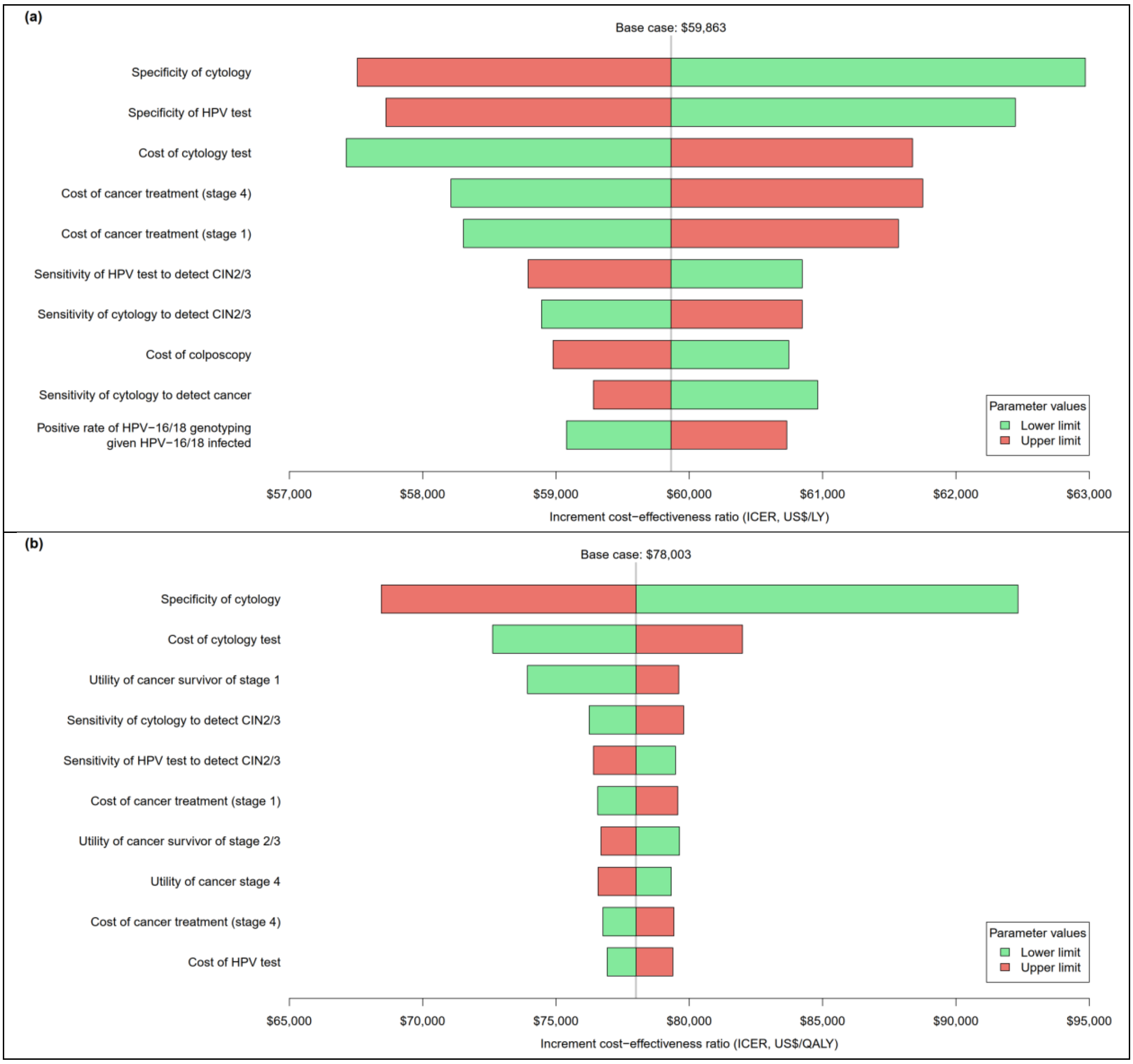

Abbreviations: CIN, cervical intraepithelial neoplasia; LY, life year; QALY, quality-adjusted life year.

Table S12. Cost-effectiveness of guidelines-based screening strategies for cohorts with the routine vaccination program (vaccinated cohorts) when 9vHPV vaccines provided lifelong protection, the vaccine uptake was 85%, and the screening uptake was 70%, with the assumption of faster HPV clearance rate and waning rate of natural immunity in males.

	Total cost, LY and QALY<br>Mean (95% percentile interval)			Difference compared with strategy A1 (Cyto-only)<br>Mean (95% percentile interval)			Incremental CEA (compared with the previous non-dominated strategy)	
Screening strategies	Total cost [a] (US$'M)	Total LY [a]	Total QALY [a]	Total cost [a] (US$'M)	Total LY [a]	Total QALY [a]	ICER per LY (US$) [b]	ICER per QALY (US$) [b]
B2 (HPV + Geno)	8.0<br>(6.8, 9.1)	297,129<br>(297,121, 297,136)	297,097<br>(297,078, 297,113)	-0.7<br>(-1.7, 0.3)	0.01<br>(-0.24, 0.35)	-8.4<br>(-17.8, -0.6)	60,229	ExtDom
B1 (HPV + Cyto)	8.3<br>(7.0, 9.4)	297,129<br>(297,121, 297,136)	297,096<br>(297,077, 297,112)	-0.4<br>(-1.4, 0.5)	0.16<br>(-0.07, 0.50)	-9.1<br>(-18.5, -1.2)	1,636,133	ExtDom
B3 (HPV + Geno and Cyto)	8.4<br>(7.1, 9.6)	297,129<br>(297,121, 297,136)	297,095<br>(297,075, 297,112)	-0.3<br>(-1.3, 0.7)	0.17<br>(-0.07, 0.50)	-10.5<br>(-20.7, -2.3)	ExtDom	ExtDom
A1 (Cyto-only)	8.7<br>(7.1, 10.1)	297,129<br>(297,121, 297,136)	297,105<br>(297,089, 297,118)	-	-	-	Dom	79,362
A2 (Cyto + Reflex HPV)	8.7<br>(7.1, 10.1)	297,129<br>(297,121, 297,136)	297,104<br>(297,087, 297,118)	0.02<br>(-0.05, 0.09)	0.16<br>(-0.07, 0.41)	-1.1<br>(-2.2, -0.2)	Dom	Dom
C1 (Co-test + Repeat cyto)	9.4<br>(7.8, 10.6)	297,129<br>(297,121, 297,136)	297,086<br>(297,064, 297,107)	0.7<br>(0.035, 1.30)	0.26<br>(0.002, 0.57)	-18.7<br>(-29.9, -7.5)	ExtDom	Dom
C2 (Co-test + Repeat co-test)	9.4<br>(7.9, 10.8)	297,129<br>(297,121, 297,136)	297,081<br>(297,055, 297,104)	0.8<br>(0.017, 1.53)	0.33<br>(0.02, 0.73)	-24.3<br>(-37.8, -10.2)	7,066,728	Dom
C3 (Co-test + Geno)	9.6<br>(8.0, 10.9)	297,129<br>(297,121, 297,136)	297,079<br>(297,053, 297,103)	0.9<br>(0.1, 1.7)	0.33<br>(0.02, 0.73)	-25.8<br>(-40.4, -10.9)	70,104,106	Dom

Abbreviations: Cyto: cytology; Dom, dominated; ExtDom, extendedly dominated; Geno, HPV-16/18 genotyping; LY, life year; QALY, quality-adjusted life year.
Assumption: The findings are based on the PSA with the parameter sets that are fitted to empirical data under the assumption that the HPV clearance rate and waning rate of natural immunity in males were 2 times faster than that in females for all HPV classes.
Notes. [a] Total cost, LY, and QALY, and corresponding differences compared with A1 (cytology-only) are presented per 10,000 individuals. Means and 95% percentile intervals are obtained based on simulation. [b] ICERs are expressed as the incremental mean cost divided by the incremental mean LY or mean QALY correspondingly, compared with the previous non-dominated strategy. The ICERs of the first non-dominated strategy are compared with no screening. ICERs that are below the WTP threshold at 1 GDPpc (US$47,792) are underlined. A strategy is dominated (Dom) if it has higher costs and worse outcomes than an alternative strategy. A strategy is extendedly dominated (ExtDom) if the ICER for the strategy is higher than that of the next more effective, non-dominated alternative strategy.

Table S13. Sensitivity analysis of the estimated cost and health outcomes of guidelines-based screening strategies for cohorts with the routine vaccination program (vaccinated cohorts) when 9vHPV vaccines provided lifelong protection, the vaccine uptake was 85%, and the screening uptake was (a) 50% and (b) 100%.

(a) Screening uptake at 50%	Total cost, LY and QALY<br>Mean (95% percentile interval)			Difference compared with strategy A1 (Cyto-only)<br>Mean (95% percentile interval)			Incremental CEA (compared with the previous non-dominated strategy)	
Screening strategies	Total cost [a] (US$'M)	Total LY [a]	Total QALY [a]	Total cost [a] (US$'M)	Total LY [a]	Total QALY [a]	ICER per LY (US$) [b]	ICER per QALY (US$) [b]
B2 (HPV + Geno)	6.4<br>(5.5, 7.2)	297,101<br>(297,082, 297,114)	297,075<br>(297,053, 297,093)	-0.4<br>(-1.2, 0.2)	0.03<br>(-0.23, 0.25)	-6.2<br>(-13.4, -0.5)	59,863	ExtDom
B1 (HPV + Cyto)	6.6<br>(5.7, 7.4)	297,101<br>(297,083, 297,114)	297,075<br>(297,052, 297,092)	-0.3<br>(-1.0, 0.4)	0.13<br>(-0.03, 0.41)	-6.8<br>(-13.9, -1.0)	1,846,885	ExtDom
B3 (HPV + Geno and Cyto)	6.7<br>(5.7, 7.5)	297,101<br>(297,083, 297,114)	297,074<br>(297,051, 297,091)	-0.2<br>(-0.9, 0.5)	0.13<br>(-0.04, 0.40)	-7.8<br>(-15.5, -1.7)	ExtDom	ExtDom
A1 (Cyto-only)	6.9<br>(5.7, 7.9)	297,101<br>(297,082, 297,114)	297,082<br>(297,061, 297,096)	-	-	-	Dom	78,003
A2 (Cyto + Reflex HPV)	6.9<br>(5.8, 7.9)	297,101<br>(297,083, 297,114)	297,081<br>(297,060, 297,096)	0.016<br>(-0.036, 0.061)	0.13<br>(-0.03, 0.35)	-0.7<br>(-1.5, -0.1)	Dom	Dom
C1 (Co-test + Repeat cyto)	7.4<br>(6.2, 8.3)	297,102<br>(297,083, 297,114)	297,068<br>(297,045, 297,086)	0.5<br>(0.020, 0.9)	0.18<br>(-0.04, 0.46)	-13.4<br>(-21.4, -5.3)	ExtDom	Dom
C2 (Co-test + Repeat co-test)	7.4<br>(6.3, 8.4)	297,102<br>(297,083, 297,114)	297,064<br>(297,039, 297,084)	0.6<br>(0.015, 1.1)	0.23<br>(0.0007, 0.56)	-17.6<br>(-27.2, -7.3)	8,589,436	Dom
C3 (Co-test + Geno)	7.5<br>(6.4, 8.5)	297,102<br>(297,083, 297,114)	297,063<br>(297,038, 297,084)	0.7<br>(0.1, 1.3)	0.23<br>(0.0007, 0.56)	-18.7<br>(-29.1, -7.8)	Dom	Dom

(b) Screening uptake at 100%	Total cost, LY and QALY<br>Mean (95% percentile interval)			Difference compared with strategy A1 (Cyto-only)<br>Mean (95% percentile interval)			Incremental CEA (compared with the previous non-dominated strategy)	
Screening strategies	Total cost [a] (US$'M)	Total LY [a]	Total QALY [a]	Total cost [a] (US$'M)	Total LY [a]	Total QALY [a]	ICER per LY (US$) [b]	ICER per QALY (US$) [b]
B2 (HPV + Geno)	10.5<br>(8.8, 12.1)	297,169<br>(297,168, 297,170)	297,127<br>(297,106, 297,145)	-0.9<br>(-2.4, 0.5)	0.06<br>(-0.45, 0.51)	-12.5<br>(-26.8, -1.1)	59,863	ExtDom
B1 (HPV + Cyto)	10.8<br>(9.0, 12.5)	297,169<br>(297,168, 297,170)	297,126<br>(297,105, 297,144)	-0.5<br>(-2.0, 0.8)	0.26<br>(-0.07, 0.81)	-13.5<br>(-27.8, -2.0)	1,846,885	ExtDom
B3 (HPV + Geno and Cyto)	11.0<br>(9.2, 12.7)	297,169<br>(297,168, 297,170)	297,124<br>(297,101, 297,143)	-0.4<br>(-1.9, 1.0)	0.26<br>(-0.07, 0.81)	-15.6<br>(-31.0, -3.5)	ExtDom	ExtDom
A1 (Cyto-only)	11.4<br>(9.1, 13.4)	297,169<br>(297,168, 297,170)	297,140<br>(297,124, 297,152)	-	-	-	Dom	78,003
A2 (Cyto + Reflex HPV)	11.4<br>(9.2, 13.4)	297,169<br>(297,168, 297,170)	297,138<br>(297,122, 297,151)	0.03<br>(-0.07, 0.12)	0.26<br>(-0.07, 0.71)	-1.5<br>(-3.1, -0.1)	Dom	Dom
C1 (Co-test + Repeat cyto)	12.4<br>(10.1, 14.2)	297,170<br>(297,168, 297,170)	297,113<br>(297,086, 297,138)	1.0<br>(0.04, 1.9)	0.37<br>(-0.08, 0.91)	-26.8<br>(-42.8, -10.6)	ExtDom	Dom
C2 (Co-test + Repeat co-test)	12.5<br>(10.3, 14.4)	297,170<br>(297,169, 297,170)	297,104<br>(297,073, 297,134)	1.1<br>(0.03, 2.3)	0.45<br>(0.0007, 1.11)	-35.1<br>(-54.5, -14.7)	8,589,436	Dom
C3 (Co-test + Geno)	12.7<br>(10.5, 14.7)	297,170<br>(297,169, 297,170)	297,102<br>(297,069, 297,133)	1.3<br>(0.2, 2.5)	0.45<br>(0.0007, 1.12)	-37.3<br>(-58.2, -15.7)	Dom	Dom

Abbreviations: Cyto: cytology; Dom, dominated; ExtDom, extendedly dominated; Geno, HPV-16/18 genotyping; LY, life year; QALY, quality-adjusted life year.
Notes. [a] Total cost, LY, and QALY, and corresponding differences compared with A1 (cytology-only) are presented per 10,000 individuals. Means and 95% percentile intervals are obtained based on simulation. [b] ICERs are expressed as the incremental mean cost divided by the incremental mean LY or mean QALY correspondingly, compared with the previous non-dominated strategy. The ICERs of the first non-dominated strategy are compared with no screening. ICERs that are below the WTP threshold at 1 GDPpc (US$47,792) are underlined. A strategy is dominated (Dom) if it has higher costs and worse outcomes than an alternative strategy. A strategy is extendedly dominated (ExtDom) if the ICER for the strategy is higher than that of the next more effective, non-dominated alternative strategy.

Table S14. One-way sensitivity analysis on the cost-effectiveness of variants of strategy B2 (HPV + Genotyping) with (a) a longer routine screening interval, (b) an older age to start screening, and (c) a predetermined number of lifetime normal HPV tests for cohorts implemented with the routine vaccination program (vaccinated cohorts) when 9vHPV vaccines provided lifelong protection, the vaccine uptake was 85%, and the screening uptake was 70%.

Strategy B2 (HPV + Genotyping)			Total cost, LY and QALY Mean (95% percentile interval)			Difference compared with strategy A1 (Cyto-only) Mean (95% percentile interval)			Incremental CEA (compared with the previous non-dominated strategy)	
Routine screening interval (year)	Start screening age (year) [a]	Lifetime normal HPV tests [b]	Total cost [c] (US$'M)	Total LY [c]	Total QALY [c]	Total cost [c] (US$'M)	Total LY [c]	Total QALY [c]	ICER per LY (US$) [d]	ICER per QALY (US$) [d]
(a) Longer routine screening interval										
15	25	NA	5.9 (5.1, 6.6)	297,125 (297,111, 297,134)	297,103 (297,087, 297,116)	-2.7 (-3.7, -1.9)	-3.23 (-6.89, -1.21)	-1.4 (-7.3, 4.6)	38,909	44,869
10	25	NA	6.4 (5.5, 7.3)	297,127 (297,115, 297,135)	297,103 (297,087, 297,116)	-2.2 (-3.2, -1.3)	-1.11 (-2.60, -0.24)	-2.0 (-8.2, 3.8)	248,372	Dom
5 [e]	25	NA	8.0 (6.8, 9.1)	297,129 (297,116, 297,136)	297,096 (297,077, 297,112)	-0.6 (-1.7, 0.3)	0.04 (-0.32, 0.36)	-8.7 (-18.8, -0.7)	1,379,922	Dom
(b) Older start screening age										
5	35	NA	5.3 (4.6, 6.0)	297,115 (297,098, 297,126)	297,093 (297,072, 297,108)	-3.4 (-4.8, -2.1)	-13.52 (-18.73, -9.34)	-11.9 (-22.7, -1.8)	35,763	42,189
5	30	NA	6.2 (5.3, 7.1)	297,125 (297,112, 297,133)	297,097 (297,078, 297,113)	-2.5 (-3.9, -1.2)	-3.83 (-6.22, -2.21)	-7.8 (-19.2, 1.8)	93,726	222,253
5 [e]	25	NA	8.0 (6.8, 9.1)	297,129 (297,116, 297,136)	297,096 (297,077, 297,112)	-0.6 (-1.7, 0.3)	0.04 (-0.32, 0.36)	-8.7 (-18.8, -0.7)	481,367	Dom
(c) Predeterimened number of lifetime normal HPV tests										
5	25	2	5.9 (5.1, 6.6)	297,121 (297,104, 297,131)	297,098 (297,079, 297,113)	-2.8 (-3.7, -1.9)	-7.89 (-14.08, -3.24)	-6.6 (-15.4, 1.0)	40,366	47,266
5	25	3	6.4 (5.5, 7.3)	297,125 (297,110, 297,134)	297,100 (297,081, 297,114)	-2.2 (-3.2, -1.3)	-3.71 (-6.84, -1.44)	-5.3 (-12.7, 1.2)	140,638	453,827
5	25	4	6.9 (6.0, 7.9)	297,127 (297,114, 297,135)	297,099 (297,081, 297,114)	-1.7 (-2.7, -0.8)	-1.55 (-3.23, -0.46)	-5.5 (-13.4, 0.9)	232,318	Dom
5	25	5	7.4 (6.3, 8.4)	297,128 (297,115, 297,136)	297,098 (297,080, 297,113)	-1.3 (-2.3, -0.4)	-0.52 (-1.21, -0.01)	-6.5 (-15.2, 0.4)	471,152	Dom
5 [e]	25	NA	8.0 (6.8, 9.1)	297,129 (297,116, 297,136)	297,096 (297,077, 297,112)	-0.6 (-1.7, 0.3)	0.04 (-0.32, 0.36)	-8.7 (-18.8, -0.7)	1,150,114	Dom

Abbreviations: Dom, dominated; LY, life year; NA, not applicable; QALY, quality-adjusted life year.

Note. [a] Following HKCOG guidelines, for strategy B2 (HPV + Genotyping), women would start screening with cytology at the age of 25 years and then switch to primary HPV test after 30 years. For strategy B2 which starts screening at the ages of 30 and 35 years, women would directly undergo primary HPV testing at 30 and 35 years, respectively, without prior cytology screening at age 25–29 years. [b] Following HKCOG guidelines, women are recommended to continue screening until age 65 years. Stopping screening after a predetermined number of lifetime normal screens would be labeled as ‘not applicable’ (NA). [c] Total cost, LY, and QALY, and corresponding differences compared with A1 (cytology-only) are presented per 10,000 individuals. Means and 95% percentile intervals are obtained based on simulation. [d] ICERs are expressed as the incremental mean cost divided by the incremental mean LY or mean QALY correspondingly, compared with the previous non-dominated strategy. The ICERs of the first non-dominated strategy are compared with no screening. ICERs that are below the WTP threshold at 1 GDPpc (US$47,792) are underlined and highlighted. A strategy is dominated (Dom) if it has higher costs and worse outcomes than an alternative strategy. [e] This indicates the initial guidelines-based strategy B2 (HPV + Genotyping) that women start cytology screening at age 25 years, then switch to primary HPV test at 30 years old with a 5-year routine screening interval, and continue to screen under age 65 years upon normal screens.

Table S15. Cost-effectiveness of variants of strategy B2 (HPV + Genotyping) for cohorts implemented with the routine vaccination program (vaccinated cohorts) when 9vHPV vaccines provided lifelong protection, the vaccine uptake was 85%, and the screening uptake was 70%.

B2 (HPV + Genotyping)			Total cost, LY and QALY<br>Mean (95% percentile interval)			Incremental CEA (compared with the previous non-dominated strategy)	
Routine screening interval (year)	Start screening age (year) [a]	Lifetime normal HPV tests [b]	Total cost [c] (US$'M)	Total LY [c]	Total QALY [c]	ICER per LY (US$) [d]	ICER per QALY (US$) [d]
10	35	2	3.4 (3.1, 3.7)	297,110 (297,092, 297,122)	297,097 (297,076, 297,111)	13,994	14,684
5	35	2	3.5 (3.2, 3.9)	297,109 (297,091, 297,121)	297,095 (297,074, 297,110)	Dom	Dom
15	30	2	3.6 (3.2, 3.9)	297,116 (297,100, 297,128)	297,102 (297,083, 297,115)	24,584	32,469
10	30	2	3.7 (3.3, 4.1)	297,117 (297,100, 297,128)	297,101 (297,082, 297,115)	ExtDom	Dom
10	35	NA	3.8 (3.4, 4.2)	297,112 (297,095, 297,124)	297,097 (297,078, 297,111)	Dom	Dom
5	30	2	3.8 (3.4, 4.2)	297,114 (297,095, 297,127)	297,097 (297,076, 297,113)	Dom	Dom
10	30	NA	3.9 (3.5, 4.3)	297,119 (297,104, 297,129)	297,102 (297,084, 297,116)	154,040	420,947
5	35	3	4.1 (3.6, 4.5)	297,112 (297,094, 297,124)	297,096 (297,075, 297,110)	Dom	Dom
5	30	3	4.4 (3.9, 5.0)	297,119 (297,103, 297,130)	297,100 (297,080, 297,114)	ExtDom	Dom
10	30	NA	4.5 (3.9, 5.0)	297,122 (297,108, 297,131)	297,103 (297,085, 297,116)	167,930	1,313,703
5	35	4	4.5 (4.0, 5.1)	297,114 (297,096, 297,125)	297,095 (297,075, 297,110)	Dom	Dom
5	35	5	4.9 (4.3, 5.6)	297,115 (297,097, 297,126)	297,094 (297,074, 297,109)	Dom	Dom
5	30	4	5.0 (4.3, 5.6)	297,122 (297,108, 297,131)	297,100 (297,081, 297,115)	ExtDom	Dom
5	35	NA	5.3 (4.6, 6.0)	297,115 (297,098, 297,126)	297,093 (297,072, 297,108)	Dom	Dom
5	30	5	5.4 (4.7, 6.2)	297,124 (297,111, 297,132)	297,099 (297,080, 297,114)	ExtDom	Dom
5	25	2	5.9 (5.1, 6.6)	297,121 (297,104, 297,131)	297,098 (297,079, 297,113)	Dom	Dom
15	25	NA	5.9 (5.1, 6.6)	297,125 (297,111, 297,134)	297,103 (297,087, 297,116)	ExtDom	2,552,055
5	30	NA	6.2 (5.3, 7.1)	297,125 (297,112, 297,133)	297,097 (297,078, 297,113)	Dom	Dom
10	25	NA	6.4 (5.5, 7.3)	297,127 (297,115, 297,135)	297,103 (297,087, 297,116)	363,119	Dom
5	25	3	6.4 (5.5, 7.3)	297,125 (297,110, 297,134)	297,100 (297,081, 297,114)	Dom	Dom
5	25	4	6.9 (6.0, 7.9)	297,127 (297,114, 297,135)	297,099 (297,081, 297,114)	Dom	Dom
5	25	5	7.4 (6.3, 8.4)	297,128 (297,115, 297,136)	297,098 (297,080, 297,113)	ExtDom	Dom
5 [e]	25	NA	8.0 (6.8, 9.1)	297,129 (297,116, 297,136)	297,096 (297,077, 297,112)	1,379,922	Dom

Abbreviations: Dom, dominated; LY, life year; NA, not applicable; QALY, quality-adjusted life year.

Note. [a] Following HKCOG guidelines, for strategy B2 (HPV + Genotyping), women would start screening with cytology at the age of 25 years and then switch to primary HPV test after 30 years. For strategy B2 which starts screening at the ages of 30 and 35 years, women would directly undergo primary HPV testing at 30 and 35 years, respectively, without prior cytology screening at age 25–29 years. [b] Following HKCOG guidelines, women are recommended to continue screening until age 65 years. Stopping screening after a predetermined number of lifetime normal screens would be labeled as ‘not applicable’ (NA). [c] Total cost, LY and QALY are presented per 10,000 individuals. Means and 95% percentile intervals are obtained based on simulation. [d] ICERs are expressed as the incremental mean cost divided by the incremental mean LY or mean QALY correspondingly, compared with the previous non-dominated strategy. The ICERs of the first non-dominated strategy are compared with no screening. ICERs that are below the WTP threshold at 1 GDPpc (US$47,792) are underlined and highlighted. A strategy is dominated (Dom) if it has higher costs and worse outcomes than an alternative strategy. A strategy is extendedly dominated (ExtDom) if the ICER for the strategy is higher than that of the next more effective, non-dominated alternative. [e] This indicates the initial guidelines-based strategy B2 (HPV + Genotyping) that women start cytology screening at age 25 years, then switch to primary HPV test at 30 years old with a 5-year routine screening interval, and continue to screen under age 65 years upon normal screens.
