# Supplementary material for: Maximizing the cost-effectiveness of cervical screening in the context of routine HPV vaccination by optimizing screening strategies with respect to vaccine uptake: a modeling analysis
Source: BMC Med. 2023 Feb 10;21:48. doi: 10.1186/s12916-023-02748-3 (PMC9921628; doi:10.1186/s12916-023-02748-3)
Supplement: Supplementary file 2 — Additional file 2: CHEERS 2022 and HPV-FRAME checklists. [file 12916_2023_2748_MOESM2_ESM.pdf]

Additional file 2: CHEERS 2022 and HPV-FRAME checklists

Consolidated Health Economic Evaluation Reporting Standards (CHEERS) 2022 Checklist

Topic	No.	Item	Location where item is reported
**Title**			
	1	Identify the study as an economic evaluation and specify the interventions being compared.	Title page
**Abstract**			
	2	Provide a structured summary that highlights context, key methods, results, and alternative analyses.	Abstract page
**Introduction**			
**Background and objectives**	3	Give the context for the study, the study question, and its practical relevance for decision making in policy or practice.	Introduction
**Methods**			
**Health economic analysis plan**	4	Indicate whether a health economic analysis plan was developed and where available.	Cost-effectiveness analysis section
**Study population**	5	Describe characteristics of the study population (such as age range, demographics, socioeconomic, or clinical characteristics).	Cervical screening section
**Setting and location**	6	Provide relevant contextual information that may influence findings.	Methods section
**Comparators**	7	Describe the interventions or strategies being compared and why chosen.	Cost-effectiveness analysis section
**Perspective**	8	State the perspective(s) adopted by the study and why chosen.	Cost-effectiveness analysis section
**Time horizon**	9	State the time horizon for the study and why appropriate.	Cost-effectiveness analysis section
**Discount rate**	10	Report the discount rate(s) and reason chosen.	Cost-effectiveness analysis section
**Selection of outcomes**	11	Describe what outcomes were used as the measure(s) of benefit(s) and harm(s).	Cost-effectiveness analysis section
**Measurement of outcomes**	12	Describe how outcomes used to capture benefit(s) and harm(s) were measured.	Cost-effectiveness analysis section
**Valuation of outcomes**	13	Describe the population and methods used to measure and value outcomes.	Cost-effectiveness analysis section

Topic	No.	Item	Location where item is reported
**Measurement and valuation of resources and costs**	14	Describe how costs were valued.	Cost-effectiveness analysis section
**Currency, price date, and conversion**	15	Report the dates of the estimated resource quantities and unit costs, plus the currency and year of conversion.	Cost-effectiveness analysis section
**Rationale and description of model**	16	If modelling is used, describe in detail and why used. Report if the model is publicly available and where it can be accessed.	Methods section and Supplementary
**Analytics and assumptions**	17	Describe any methods for analysing or statistically transforming data, any extrapolation methods, and approaches for validating any model used.	Methods section and Supplementary
**Characterising heterogeneity**	18	Describe any methods used for estimating how the results of the study vary for subgroups.	NA
**Characterising distributional effects**	19	Describe how impacts are distributed across different individuals or adjustments made to reflect priority populations.	NA
**Characterising uncertainty**	20	Describe methods to characterise any sources of uncertainty in the analysis.	Vaccination, Cervical screening, Cost-effectiveness section
**Approach to engagement with patients and others affected by the study**	21	Describe any approaches to engage patients or service recipients, the general public, communities, or stakeholders (such as clinicians or payers) in the design of the study.	NA
**Results**			
**Study parameters**	22	Report all analytic inputs (such as values, ranges, references) including uncertainty or distributional assumptions.	Table 3 and Supplementary
**Summary of main results**	23	Report the mean values for the main categories of costs and outcomes of interest and summarise them in the most appropriate overall measure.	All paragraphs in Results
**Effect of uncertainty**	24	Describe how uncertainty about analytic judgments, inputs, or projections affect findings. Report the effect of choice of discount rate and time horizon, if applicable.	Table 3, Figure 3, Table S6-S8, Table S10-S14

Topic	No.	Item	Location where item is reported
**Effect of engagement with patients and others affected by the study**	25	Report on any difference patient/service recipient, general public, community, or stakeholder involvement made to the approach or findings of the study	NA
**Discussion**			
**Study findings, limitations, generalisability, and current knowledge**	26	Report key findings, limitations, ethical or equity considerations not captured, and how these could affect patients, policy, or practice.	Discussion
**Other relevant information**			
**Source of funding**	27	Describe how the study was funded and any role of the funder in the identification, design, conduct, and reporting of the analysis	Funding section
**Conflicts of interest**	28	Report authors conflicts of interest according to journal or International Committee of Medical Journal Editors requirements.	Declarations section

Note: Referred to Consolidated Health Economic Evaluation Reporting Standards (CHEERS) 2022 Checklist.[88]

1 HPV-FRAME reporting standard checklist

Items	Reported? (Y / N)	Reported by age? (Y / N)	Reported by sex? (F-only, M-only, or Both)	Comments
(a) Inputs				
Target population for intervention	Y	Y	F	Methods.
Sexual behaviour	Y	Y	B	Supplementary.
Cohort examined for evaluation/time horizon	Y (multiple cohorts)	Y	F	Methods: Lifetime outcomes among women in unvaccinated cohorts and women in the first 10 years in vaccinated cohorts considered.
Quality of life assumptions	Y	N	F	Methods and Supplementary. Health utilities for attending screenings and receiving treatment were considered when calculating QALYs.
Calibration	Y	Y	F	The model was calibrated to pre-vaccination HPV prevalence and cervical cancer incidence.
Validation (where possible)	N	N/A	N/A	
Costs	Y	N	N	Methods and Supplementary.
Routine screening behaviour (routine and follow-up and test-of-cure)	Y	Y	F	Methods and Supplementary. Tests for cervical screening may change according to evaluated strategies.
Screening test(s) and colposcopy accuracies	Y	N (invariant within age range)	F	Methods and Supplementary.
Abnormal test management (primary and triage)	Y	N (invariant within age range)	F	Methods and Supplementary. Management for screening strategies was based on guidelines recommended by local healthcare authorities.<br>Four cytology results were considered: normal, ASCUS, LSIL and HSIL.
Diagnostic follow-up of abnormal tests	Y	N (invariant within age range)	F	Methods and Supplementary. Management for screening strategies was based on guidelines recommended by local healthcare authorities.
Management by disease grade (confirmed disease)	Y	N (invariant within age range)	F	Methods and Supplementary. Management for screening strategies was based on guidelines recommended by local healthcare authorities.
Sources of information for screening structure and parameterization	Y	Y	F	Methods and Supplementary. Management for screening strategies was based on guidelines recommended by local healthcare authorities.
Vaccine uptake	Y	Y	F	Methods: vaccine uptake among women in vaccinated cohorts was based on statistics in an immunization program and by assumed scenarios, and that in unvaccinated cohorts was based on local surveys.

Items	Reported? (Y / N)	Reported by age? (Y / N)	Reported by sex? (F-only, M-only, or Both)	Comments
Vaccine efficacy	Y	N	F	Methods: efficacy is assumed to be the same across ages.
Vaccine cross-protection	Y	N	N	Methods: efficacy is assumed to apply against vaccine-targeted types only (no cross-protection).
Duration of vaccine protection and waning	Y	N	F	Method: Scenarios of assumed and fixed durations of vaccine protection.
Vaccine and delivery costs	N	N	N	Methods: The model evaluates the impact of cervical screening on top of routine HPV vaccination which has been included in an immunization program.
Pre-vaccination disease burden (including PAFs)	N	N	N	Not reported explicitly, but accounted for when calculating QALYs.
Heterogeneity in sexual behaviour	Y	Y	B	Supplementary: Assortativeness of sexual mixing by age and by sexual activity levels were parametrized via model calibration; proportions in sexual activity levels by age groups were based on local surveys.
Duration of natural immunity	Y	N	N	Supplementary: Natural immunity by HPV types based on calibration (exponential waning) and was assumed to be the same across age and genders.
Herd effect	Y	Y	Y	Method and supplementary: Herd effect was accounted for in the dynamic model.
Association between vaccination and cervical screening	Y	N	N	Methods: Assumed independence between vaccination and cervical screening.
(b) Outputs				
Cancer incidence, mortality, life years, QALYs/DALYs (as appropriate)	Y	Y (implicitly)	F	Results: QALYs and LYs reported.
HPV prevalence, pre-intervention	N	N/A	N/A	Not reported explicitly, but accounted for in model calibration and when calculating QALYs.
CIN2/3 detected	N	N/A	N/A	Not reported explicitly, but accounted for when calculating QALYs.
Sensitivity analysis on key inputs	Y	N	N	Results: by vaccine uptake, duration of vaccine protection and discount rate in one-way sensitivity. Parameters of natural history and health economics were considered in the probabilistic sensitivity analysis.
Incremental cost-effectiveness ratios and costs saved	Y	Y (implicitly)	F	Results: Tables.
Absolute reductions in HPV infections, cervical and other HPV-related cancers and/or warts, post-vaccination	N	N/A	N/A	Not reported explicitly, but accounted for when calculating QALYs.

Items	Reported? (Y / N)	Reported by age? (Y / N)	Reported by sex? (F-only, M-only, or Both)	Comments
Absolute reductions in CIN2+ post-vaccination	N	N/A	N/A	Not reported explicitly, but accounted for when calculating QALYs.
Absolute reductions in invasive cancer post-vaccination	N	N/A	N/A	Not reported explicitly, but accounted for when calculating QALYs.

Note: Referred to HPV-FRAME reporting standard checklist.[89]

Abbreviations: B: both female and male; CIN: cervical intraepithelial neoplasia; F: female-only; HSIL: high-grade squamous intraepithelial lesion; LSIL: low-grade squamous intraepithelial lesion; LY: life year; M: male-only; N: no; N/A: not applicable; PAF: population attributable fraction; QALY: quality-adjusted life year; Y: yes.
